# Pathogenic Significance of Trypanosomatids: Progress in Drug Resistance, Control Strategies, and Artificial Intelligence

**DOI:** 10.1155/ipid/7565247

**Published:** 2026-06-28

**Authors:** Cynthia Mmalebna Amisigo, Aboagye Kwarteng Dofuor, Jennifer Afua Afrifa Yamoah, Albert Fynn Aiduenu, Blessing Kwabena Gayi, Angelina Akomea, Zenabu Lansah Alidu, Gideon Danso, Selina Mawunyo Ayivi-Tosuh, Naa Kwarley-Aba Quartey, Joseph Harold Nyarko Osei, Seyram Kofi Loh, William Ekloh

**Affiliations:** ^1^ West African Centre for Cell Biology of Infectious Pathogens, University of Ghana, Legon, Accra, Ghana, ug.edu.gh; ^2^ Department of Biochemistry, Cell and Molecular Biology, University of Ghana, Legon, Accra, Ghana, ug.edu.gh; ^3^ Department of Biological Sciences, School of Natural and Environmental Sciences, University of Environment and Sustainable Development, Somanya, Ghana, uesd.edu.gh; ^4^ Animal Health Division, Animal Research Institute, Council for Scientific and Industrial Research, Adenta-Frafraha, Accra, Ghana, csir.co.za; ^5^ Department of Pharmaceutical Sciences, University of Oklahoma Health, Oklahoma City, Oklahoma, USA; ^6^ Department of Molecular Pharmaceutics, University of Utah, Salt Lake City, Utah, USA, utah.edu; ^7^ Department of Biomedical Sciences, University of Cape Coast, Cape Coast, Ghana, ucc.edu.gh; ^8^ Department of Food Science and Technology, Ho Technical University, Ho, Ghana, htu.edu.gh; ^9^ Department of Wood Science and Technology, College of Agriculture and Renewable Natural Resources, Kwame Nkrumah University of Science and Technology, Kumasi, Ghana, knust.edu.gh; ^10^ Biomedical and Public Health Research Unit, Water Research Institute, Council for Scientific and Industrial Research, Accra, Ghana, csir.co.za; ^11^ Department of Parasitology, Noguchi Memorial Institute for Medical Research, College of Health Sciences, University of Ghana, Legon, Accra, Ghana, ug.edu.gh; ^12^ Department of Built Environment, School of Sustainable Development, University of Environment and Sustainable Development, Somanya, Ghana, uesd.edu.gh; ^13^ Department of Biochemistry, School of Biological Sciences, University of Cape Coast, Cape Coast, Ghana, ucc.edu.gh

**Keywords:** artificial intelligence, drug resistance, kinetoplastids, *Leishmania*, neglected tropical diseases, *Trypanosoma*

## Abstract

Trypanosomatids, including the genera *Trypanosoma* and *Leishmania*, are protozoan parasites of significant medical and veterinary relevance. These organisms cause African trypanosomiasis, Chagas disease, and leishmaniasis, which are classified as neglected tropical diseases (NTDs). NTDs continue to present major public health challenges, particularly in sub‐Saharan Africa, Latin America, and parts of Asia, resulting in substantial morbidity, mortality, and socioeconomic impacts. Chemotherapeutic interventions remain constrained by toxicity, high cost, limited accessibility, and the increasing prevalence of drug resistance. Significantly, there is a tight relationship between pathogenesis and drug resistance because processes that facilitate intracellular persistence, immunological evasion, and metabolic adaptation can also decrease drug sensitivity and increase treatment failure. This interaction makes it easier for more pathogenic and resilient strains to arise, which makes disease treatment and control more difficult. The absence of effective vaccines underscores the urgent need for novel therapeutic strategies. Advances in molecular parasitology have elucidated unique organellar structures, immune evasion mechanisms, and metabolic pathways in trypanosomatids, providing new therapeutic targets. Additionally, artificial intelligence and computational methodologies are facilitating drug discovery, predicting resistance, and improving diagnostics. In this review, we provide a narrative overview of current knowledge of trypanosomatid biology, prevalence, drug resistance mechanisms, and emerging control strategies. By integrating classical parasitology with computational approaches, new opportunities are identified to address resistance, enhance therapy, and mitigate the global health burden of trypanosomatid infections.

## 1. Introduction

Kinetoplastids are unicellular eukaryotic organisms. They are flagellated protists of the class Kinetoplastea in the phylum Euglenozoa [1, 2]. They are characterized by a kinetoplast, a nucleus‐like organelle that is a large mass of DNA within the single mitochondrion of the cell [2, 3]. Kinetoplastids encompass a diverse range of free‐living species and many that cause diseases in humans, livestock, and certain plants [1, 2, 4]. Some kinetoplastids are free‐living. Pathogenic genera such as *Trypanosoma* and *Leishmania* cause persistent and devastating neglected tropical diseases [2, 4, 5]. These parasites exert a heavy toll on health, especially in low‐resource settings, by perpetuating cycles of poverty, disability, and premature mortality. Prompt, effective treatment is essential to halt these acute conditions and improve patient outcomes.

*Trypanosoma cruzi*, the primary agent of Chagas disease, affects an estimated 6–7 million people, primarily in Latin America, where triatomine bugs serve as the primary vector of the disease [6]. Chagas disease moves from an acute stage of high parasitemia to a chronic stage that can cause fatal cardiomyopathy and severe gastrointestinal complications [7, 8]. African trypanosomes, spread by tsetse flies (*Glossina* spp.) and some biting flies, cause human African trypanosomiasis (HAT), also known as sleeping sickness. This disease presents in two forms: the chronic West and Central African form, caused by *T. brucei gambiense*, and the acute East African form, caused by *T. brucei rhodesiense* [9, 10]. Without timely treatment, HAT is always fatal. This urgency demands strong disease surveillance and effective treatments. In addition, *T. vivax, T. brucei,* and *T. congolense* are major causes of animal African trypanosomiasis (AAT), undermining livestock production and food security across sub‐Saharan Africa [11, 12].

Leishmaniasis, caused by over 20 *Leishmania* species, shows the widespread impact of kinetoplastid infections. It is endemic in over 90 countries and affects about 12 million people, with hundreds of millions at risk [13]. Its clinical spectrum ranges from self‐healing cutaneous lesions to severe mucocutaneous forms and visceral leishmaniasis (VL). The latter is fatal if untreated [14]. Transmission occurs through bites from infected phlebotomine sand flies. Zoonotic reservoirs make control harder. These diseases collectively demonstrate the challenges posed by kinetoplastid parasites. Their complex life cycles, antigenic variation, and ecological reservoirs hinder the development of effective elimination strategies.

Historically, control has relied on chemotherapy. Drugs such as benznidazole, nifurtimox, eflornithine, pentamidine, amphotericin B (AmB), and miltefosine are mainstays of treatment [15, 16]. However, these have well‐known limitations: prolonged regimens, notable toxicity, high cost, and increasing resistance in both clinical and veterinary contexts [16, 17]. The lack of effective vaccines increases reliance on chemotherapy. Antigenic variation in *Trypanosoma* and the intracellular persistence of *T. cruzi* and *Leishmania* have hindered vaccine development [18, 19]. As a result, resistance has become a significant threat, jeopardizing progress in endemic regions.

Advances in genomics, transcriptomics, and proteomics have elucidated molecular mechanisms underlying drug resistance in kinetoplastids. Alterations in drug transporters, overexpression of efflux pumps, and metabolic adaptations contribute to resistance against first‐line therapies [20, 21]. These findings enhance the understanding of parasite biology and facilitate the identification of novel molecular targets for therapeutic intervention. Concurrently, artificial intelligence (AI) and computational modeling are transforming parasitology research by enabling high‐throughput compound screening, resistance prediction, and optimization of combination therapies [22, 23]. Additionally, digital epidemiology and machine learning are improving disease surveillance, diagnostics, and the personalization of treatment strategies [24, 25].

Despite advances in understanding trypanosomatid biology, critical gaps remain in elucidating mechanisms of emerging drug resistance. Thus, there is a need to address new control methods and how AI can help improve diagnosis, monitoring, and sustained management of trypanosomatid‐associated diseases. This review aims to provide comprehensive insights into the pathogenic significance of trypanosomatids in the context of prevalence, molecular findings, and new technologies for drug resistance and control strategies. Using a One Health approach, we address human, animal, and environmental factors, emphasizing the need for integrated solutions that consider the interconnectedness of these domains. The review also highlights the potential of AI to accelerate drug discovery and enhance control frameworks. Understanding the dynamic links between parasite biology, host responses, and technology is crucial to overcoming drug resistance.

## 2. Global Distribution and Prevalence

**FIGURE 1:**
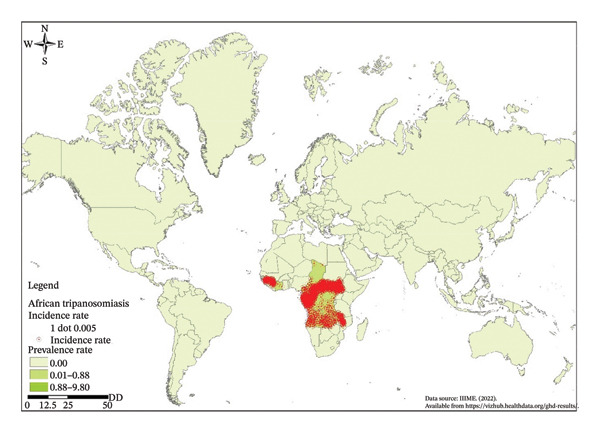
Global distribution of African trypanosomiasis incidence and prevalence rates in 2021. The prevalence (shown by green hues) is primarily concentrated in Central and East Africa, with the highest levels observed in the Democratic Republic of Congo, Angola, Central African Republic, South Sudan, and Chad. The incidence (red dots) demonstrates a concentrated clustering in specific areas, highlighting the disease’s localized endemicity and the pressing necessity for focused surveillance and vector management.

The prevalence rate of African trypanosomiasis, indicated by varying shades of color (green) (Figure 1), is predominantly found in sub‐Saharan Africa, with the greatest rates (0.88–9.80 per 100,000 people) recorded in the Democratic Republic of Congo (DRC), Angola, Central African Republic, South Sudan, Chad, and certain regions of Uganda. Reduced prevalence rates (0.01–0.88 per 100,000) extend into adjacent nations, such as Congo (Brazzaville), Cameroon, and Sudan. Incidence is represented by the density of red dots, with each dot corresponding to 0.005 cases per 100,000 individuals. The highest concentrations of incidence are found in the DRC, with significant levels also present in the Central African Republic, Angola, and South Sudan. Incidence clusters are dispersed but less intense in Chad, Uganda, and Cameroon, whereas the remainder of the world exhibits minimal or no known incidence. This regional distribution highlights the localized endemicity of African trypanosomiasis in Central and East Africa, illustrating the ecology of the tsetse fly vector and the unequal disease burden in nations with constrained healthcare resources. The map highlights the necessity of localized surveillance and vector control measures to mitigate transmission and decrease illness prevalence.

**FIGURE 2:**
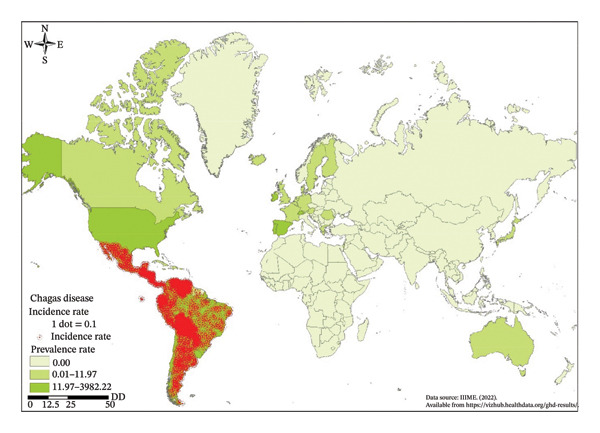
Global distribution of Chagas disease incidence and prevalence rates in 2021. High prevalence (green) and dense incidence clusters (red dots) are primarily located in Latin America, especially in Brazil, Bolivia, Argentina, and Paraguay, whereas lesser prevalence is noted in Mexico, Colombia, and Venezuela. Nonendemic nations such as the United States and Spain exhibit imported cases, indicative of transmission driven by migration.

The worldwide distribution and prevalence rate of Chagas disease, represented in varying shades of green, is predominantly concentrated in Latin America, especially in Brazil, Bolivia, Argentina, and Paraguay, where it surpasses 11.97 per 100,000 population (Figure 2). Moderate incidence is noted in Colombia, Venezuela, Mexico, and Peru, whereas nonendemic nations such as the United States, Spain, and Japan have minimal levels, indicating imported cases rather than indigenous transmission. The incidence, indicated by the red dot density (1 dot = 0.1 per 100,000), exhibits a comparable trend, with the most concentrated clusters located in Brazil, Bolivia, and Argentina and expanding into Paraguay, Peru, and Colombia. The southern sections of Chile and certain areas of Mexico exhibit dispersed yet significant incidence clusters. The occurrence outside Latin America remains minimal. This distribution emphasizes Chagas disease as a primarily Latin American public health issue, with endemic transmission facilitated by the triatomine vector.

**FIGURE 3:**
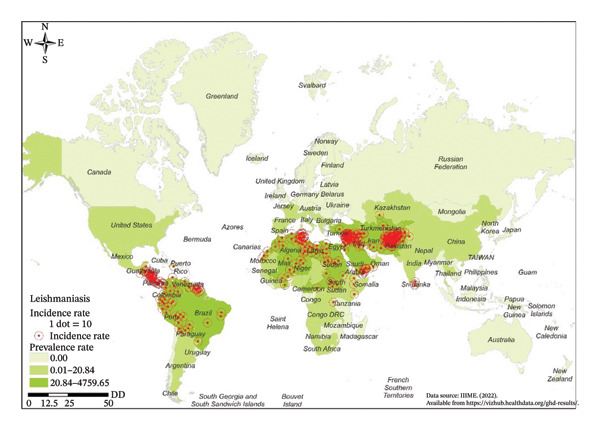
Global distribution of leishmaniasis incidence and prevalence rates in 2021. High prevalence (dark green) and concentrated incidence clusters (red dots) are mostly found in Brazil, India, Sudan, South Sudan, and Ethiopia, with supplementary hotspots throughout South America, East Africa, the Middle East, South Asia, and the Mediterranean.

The prevalence of leishmaniasis, illustrated in color (green) hues, is centered in South America, East Africa, North Africa, the Middle East, and South Asia (Figure 3). The highest incidence rates (> 20.84 per 100,000 population) are found in Brazil, Paraguay, Venezuela, Peru, and Colombia in South America; Sudan, South Sudan, Ethiopia, Somalia, and Kenya in East Africa; and India, Nepal, and Pakistan in South Asia. However, it is worth noting that since the launch of the Kala‐azar Elimination Program (KAEP) by India, Bangladesh, and Nepal in 2005, India recently achieved the goal of kala‐azar elimination, defined as an incidence below 1 per 10,000 at subdistrict (block) level [26]. Moderate incidence is observed throughout North Africa (Morocco, Algeria, and Egypt), the Middle East (Iran, Iraq, Syria, Saudi Arabia, and Yemen), and certain regions of Central Asia (Turkmenistan and Uzbekistan). The incidence, indicated by the red dot density (1 dot = 10 per 100,000), corroborates this distribution, with the most concentrated clusters located in Brazil, India, Sudan, South Sudan, and Ethiopia. Further hotspots are evident in Turkey, Iran, Afghanistan, and Pakistan, alongside dispersed clusters in Spain, Italy, and France, indicating the Mediterranean emphasis of zoonotic cutaneous leishmaniasis (CL). This regional distribution underscores leishmaniasis as a prevalent neglected tropical disease with endemic foci in both the New World and the Old World. The concentration of high incidence and prevalence in low‐ and middle‐income areas underscores the necessity for enhanced vector control, prompt case identification, and improved access to treatment to alleviate the worldwide disease burden.

## 3. Comparative Genomics and Surface Biology of Trypanosomatids

*Leishmania major, T. brucei,* and *T. cruzi* are collectively classified as the TriTryp kinetoplastids following the landmark genomic studies by three independent teams [27–29]. Within *Leishmania*, species such as *L. donovani* and *L. major* are categorized as Old World types, whereas *L. mexicana* and *L. braziliensis* belong to the New World groups [29]. Although the TriTryps share similar biochemical compositions, metabolic pathways, and extensive orthologous gene content, notable genomic divergence exists. For instance, approximately 910 genes in *L. major* lack orthologs in *T. brucei* and *T. cruzi*, while 482 orthologous groups are shared between *L. major* and *T. cruzi*, and only 74 between *L. major* and *T. brucei* [29].

These genomic differences underpin important metabolic distinctions. *Leishmania*‐specific genes encode glycoconjugate biosynthesis components, peptidases, and transporters that differentiate them from *T. brucei* and *T. cruzi* [29]. Notably, two genes (LmjF33.1740 and LmjF33.1750) encode proteins with macrophage migration inhibitory factor (MIF)–like domains that share 30%–40% similarity with eukaryotic homologs. While retaining tautomerase activity, these proteins lack thiol oxidoreductase function, suggesting limited evolutionary divergence from ancestral bacterial forms [29]. Importantly, human MIF has been shown to activate macrophages against *Leishmania* via a T helper 1 (Th1)–type immune response [29].

Kinetoplastid signaling systems further reflect both conservation and divergence. The TriTryps possess extensive families of kinesins, adenylate cyclases, protein kinases (PKs), and phosphatases [28]. *T. brucei* exhibits a particularly expanded repertoire of adenylate cyclases (47 genes), compared to *T. cruzi* (25 genes) and *L. major* (11 genes), indicating greater signaling complexity [28]. In contrast to classical eukaryotic systems, trypanosomatid PKs lack domains such as SH2, SH3, FN III, and immunoglobulin‐like domains, and they do not possess canonical tyrosine kinases. Instead, they rely on dual‐specificity kinases and phosphoinositide metabolism pathways, emphasizing adaptations for stress response and cell cycle regulation [28]. For example, *T. brucei* utilizes multiple cyclin‐dependent kinases, cyclins, and MAP kinase pathway components for proliferation, differentiation, and flagellar control [30]. These differences highlight PKs as promising drug targets.

Surface molecule architecture plays a central role in host–parasite interactions and immune evasion. *T. brucei* relies on antigenic variation mediated by a dense coat of approximately 10^7^ copies of variant surface glycoprotein (VSG), which undergoes continuous replacement to evade host immunity [31, 32]. In contrast, *T. cruzi* and *L. major* exhibit broader surface molecule diversity. For example, *T. cruzi* dedicates hundreds of genes to encode glycoprotein 63 (gp63) proteases, mucins, trans‐sialidases (TSs), and mucin‐associated surface proteins (MASPs) [28, 33]. TS, which facilitates the acquisition of host‐derived sialic acid, exists as a large superfamily in *T. cruzi* with both active and inactive members, many containing SAPA repeats that contribute to immune evasion [34]. TS is absent in *L. major* and less prominent in *T. brucei*, reinforcing its importance as a drug target [28].

Glycosylation is a defining feature of kinetoplastid surface biology. Although these parasites possess glycosyltransferases, many cannot synthesize sialic acid de novo and instead depend on host‐derived sources [28, 35, 36]. Both *T. brucei* and *T. cruzi* utilize surface‐bound TSs to transfer sialic acid from host glycoconjugates to parasite surface molecules, specifically to procyclin GPI anchors in *T. brucei* and to mucin O‐glycans in *T. cruzi* [35]. Additionally, mucins, absent in *T. brucei* but present in *L. major*, are highly expanded in *T. cruzi*, where they are classified into TcMUC and TcSMUG subfamilies with stage‐specific expression patterns [28].

Protease systems also contribute significantly to virulence and host interaction. The gp63 metalloproteases are implicated in host cell invasion, protein shedding, and pathogenicity [37]. Strikingly, *T. cruzi* contains over 420 gp63‐related genes and pseudogenes, compared to only 13 in *T. brucei* and six (including pseudogenes) in *L. major*, raising questions about the evolutionary advantage of such expansion [28]. These surface and enzymatic adaptations collectively underscore the diverse strategies employed by TriTryps to survive within hosts.

At the mitochondrial level, apocytochrome b (CYb) plays a central role in electron transport and drug resistance. Encoded by kinetoplast DNA, CYb contains approximately 400 amino acids and eight transmembrane domains, and it binds two heme groups (b562 and b566) essential for electron transfer within the Q‐cycle [38, 39]. The Qi and Qo sites, located on opposite sides of the inner mitochondrial membrane, facilitate ubiquinone redox cycling and are critical for parasite respiration [38, 40].

Recent advances in high‐resolution transcriptomics have significantly refined our understanding of trypanosomatid biology beyond the foundational genome projects. Single‐cell RNA sequencing (scRNA‐seq) studies in *T. brucei* and *Leishmania* have revealed previously unrecognized heterogeneity within parasite populations, including intermediate developmental forms and transcriptionally distinct subpopulations that contribute to adaptation and persistence within hosts [41, 42]. These findings challenge the classical view of discrete life cycle stages and instead suggest a continuum of differentiation states regulated by environmental cues and host interactions.

In parallel, epigenetic regulation has emerged as a critical mechanism governing gene expression in trypanosomatids, which largely lack conventional transcriptional control. Chromatin remodeling, histone variants, and posttranslational modifications have been shown to regulate antigenic variation, particularly VSG expression in *T. brucei* [43]. Nuclear architecture also plays a role, with VSG expression sites localized to specialized transcriptionally active compartments, ensuring monoallelic expression and immune evasion. These discoveries highlight the importance of spatial genome organization in parasite survival.

Extracellular vesicles (EVs) have recently been identified as important mediators of host–parasite communication. Trypanosomatids release EVs containing proteins, RNA, and virulence factors that can modulate host immune responses, enhance parasite infectivity, and potentially transfer drug resistance traits between parasite populations [44, 45]. This emerging mode of intercellular communication represents a novel dimension of parasite biology with implications for pathogenesis and therapeutic targeting.

Furthermore, CRISPR‐Cas9–based functional genomics has revolutionized the study of kinetoplastids by enabling genome‐wide loss‐of‐function screens. These approaches have facilitated the identification of essential genes, novel virulence factors, and drug resistance determinants, thereby accelerating target discovery and validation [46, 47]. Together, these advances provide a more dynamic and integrated view of trypanosomatid biology.

## 4. History of Treatment and Drug Resistance

Host‐parasite relationships have been in existence for a long time. Infection and treatment of HAT became important in the middle ages [48]. The first medically accurate report of HAT was in 1734, which has helped with accurate diagnoses and treatment of HAT during several major outbreaks in the nineteenth and twentieth centuries [49–51].

In the early 1900s, trypan red and trypan blue were drugs initially used for trypanosome chemotherapy [52]. Atoxyl, an aminophenyl arsenic acid (the first of the arsenical drugs), was used for the treatment of first‐stage HAT [53]. Tryparsamide was then discovered in the 1920s for the successful treatment of second‐stage HAT [53]. Melarsen which was less toxic than the existing drugs for trypanosome treatment was discovered after tryparsamide [53, 54]. It was, however, very expensive. Melarsen oxide (MelOx) was the next synthesized derivative of melarsen. The issue of toxicity remained a challenge for the HAT drug treatment regimen. Another arsenical drug called melarsoprol was therefore synthesized in the 1940s [53–55]. This drug was a formulation that combined dimercaprol, an arsenic antidote, with MelOx [54]. Having this new drug, toxicity issues with HAT treatment regimen reduced remarkably. Melarsoprol remained the only widely used drug for HAT treatment since the 1950s. In 1929, some diamidines and their derivatives were found to have trypanocidal effects with manageable toxicity levels. Some examples include synthalin, diamidino‐1,11‐n‐undecane, stilbamidine, and pentamidine [54]. However, melarsoprol remained important for treating HAT infections for about half a century until eflornithine was produced and repurposed for second stage HAT treatment in the 1980s [53]. In the 1960s, a nitrofuran derivative called nifurtimox was discovered for the treatment of Chagas disease in Latin America [54]. In 2009, following observed drug resistance in some melarsoprol‐resistant trypanosome populations, the nifurtimox–eflornithine combination therapy (NECT) treatment regimen was introduced as a safer and simpler therapy to help control and eliminate the HAT disease [56].

Increasing reports of resistance to HAT treatment regimens had been reported since the second half of the twentieth century in East Africa [55]. The need to develop new HAT treatment regimens was partly due to the high toxicity of existing regimens. Another reason was the observation that trypanosomes circulating in endemic foci were becoming tolerant to the existing drugs for HAT treatment [15]. In the late 1990s, melasoprol resistance was reported [57]. The last two drugs used as monotherapy for HAT treatment were nifurtimox and eflornithine.

Leishmaniasis was referred to as oriental sores or a specific type of boils depending on where it was found [58, 59]. For instance, leishmaniasis found in Jericho was called Jericho boils [58]. This disease existed before the separation of Pangea in the Mesozoic era over 250 million years ago [58, 59] and has been recently confirmed by modern molecular techniques [60, 61]. Several medicines used as treatment options proved futile. Lieutenant General Sir William Boog Leishman, however, found mercurial plaster as a treatment option that worked. The genus *Leishmania* was named after him as credit for his contribution to leishmaniasis research just as Charles Donovan was credited for discovering the visceral form of the disease [59].

It is worth reiterating that there was no potent treatment regimen in antiquity for the treatment of leishmaniasis. The search for potent drugs for leishmaniasis treatment has therefore been a long journey. Several concoctions and formulations had been tried prior to the 20th century without success. Treatment for leishmaniasis dates back as far as the 1900s [58]. Similar to HAT treatment regimens, there are just a few drug options approved for the treatment of leishmaniasis [62]. Several clinical trials have also been conducted either as monotherapy or combination therapy for *Leishmania* treatment. A typical example is the several Phase II and III trials done in India leading to the approval of oral administration of miltefosine trypanosomatids infection. This was due to the independent discovery of antiprotozoal and antineoplastic properties of miltefosine and some related alkylphosphocholine drugs in the 1980s by two different research groups in Germany and the United Kingdom [63]. This has added a few drugs for the treatment of the different types of leishmaniasis. The common types of the disease are CL and VL. However, there are other forms such as the mucocutaneous leishmaniasis (MCL) and post–kala‐azar dermal leishmaniasis (PKDL) [64].

Antimonials were the first drugs discovered for the treatment of leishmaniasis. They were found in the 1920s [64]. The potential for antimonials to be used for therapeutic purposes had been documented in the 1600s and 1700s. It was referred to as one of the seven wonders of the world [65]. In 1945, a derivative of these antimonials, stibogluconate, was discovered for treatment [64]. Having a few drugs for the treatment of leishmaniasis, this increased the number of drugs for treatment. A good number of these treatment regimens happened to be repurposed drugs [66]. Examples of these are the pentavalent antimonials Sb(V), which include sodium stibogluconate, glucantime, or meglumine antimoniate; the amidine compounds such as pentamidine; the aminoglycosides such as paromomycin; alkylphosphocholine compounds called miltefosine, sitamaquine, and 8‐aminoquinoline analog; and the polyene antifungals namely AmB [63, 64, 67]. These drugs may be administered orally, intramuscularly, intravenously, subcutaneously, and topically [68].

Pentavalent antimonials were some of the drugs discovered earlier for the treatment of leishmaniasis. The antimonials have been used as a standard first‐line treatment since the 1930s till date [64, 68]. Due to issues of toxicity, it was imperative to develop alternative drugs. In the 1980s, AmB was discovered as an alternative for the treatment of leishmaniasis [58]. Various doses have also been suggested for the same as well as different forms and manifestations of leishmaniasis [65, 69]. This was largely due to the drive for the search of novel and/or nontoxic active compounds. Another reason was ascertaining effective doses for shorter treatment regimens.

Recent studies have shown that resistance among trypanosomatids remains a major obstacle to disease control despite the introduction of newer combination therapies and repurposed drugs. In *T. brucei*, reduced susceptibility to melarsoprol and pentamidine has continued to be linked to alterations in parasite transporters such as the TbAT1/P2 adenosine transporter and aquaglyceroporin 2 (AQP2), which are involved in drug uptake [57, 70]. Mutations, deletions, or chimeric rearrangements affecting these transporters have been associated with treatment failure in endemic regions. Furthermore, although NECT significantly improved treatment outcomes for HAT, laboratory and field evidence suggests that decreased sensitivity to nifurtimox and eflornithine may emerge through metabolic adaptation and transporter‐associated mechanisms [15, 71]. More recently, the introduction of the oral drug fexinidazole represented a major breakthrough in HAT treatment; however, experimental studies have already demonstrated that prolonged exposure can select resistant *T. brucei* strains through impaired nitroreductase (NTR) activity, raising concerns regarding future therapeutic sustainability [72]. These findings underscore the continuing evolutionary capacity of trypanosomes to evade chemotherapeutic pressure and highlight the importance of surveillance systems and novel drug target discovery.

Similarly, increasing resistance has become a significant concern in leishmaniasis chemotherapy, particularly in regions where antimonial drugs have been extensively used for decades. Widespread resistance to pentavalent antimonials has been reported in parts of India, Nepal, and Bangladesh, especially among *L. donovani* isolates causing VL [73, 74]. Resistance mechanisms have been associated with reduced drug uptake, enhanced efflux through ATP‐binding cassette (ABC) transporters, thiol metabolism alterations, and increased parasite antioxidant defenses [73]. Although AmB and miltefosine improved treatment success rates, emerging reports of relapse and treatment failure have also been documented. Miltefosine resistance has particularly been linked to mutations in the miltefosine transporter complex (LdMT and LdRos3), reducing intracellular drug accumulation [67, 75]. Current research therefore emphasizes integrated strategies involving combination therapies, molecular surveillance, and the development of new antiparasitic compounds to mitigate the growing threat of resistance among trypanosomatids.

## 5. Mechanisms of Drug Action and Resistance

### 5.1. Pentavalent Antimonials

**FIGURE 4:**
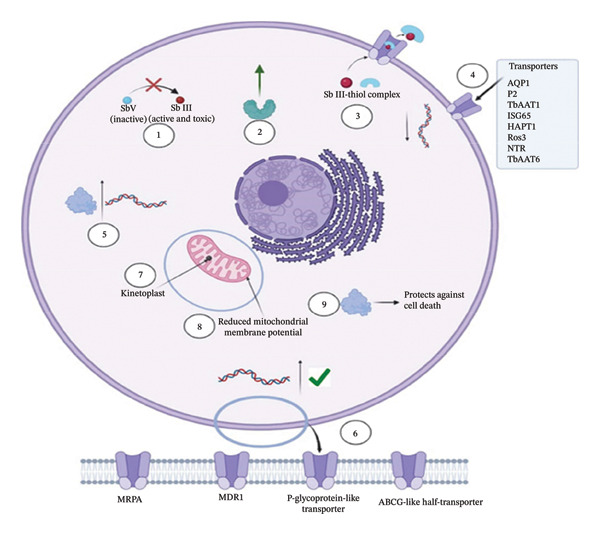
Proposed mechanisms of drug resistance in trypanosomatids. (1) Decreased conversion of inactive to active form, e.g., decreased reduction of SbV (inactive) to SbIII (active); (2) increased trypanothione production; (3) increased efflux of Sb(III)–thiol complex; (4) gene deletion or downregulation of transporters; (5) upregulation of proteins involved in translation, stress response, and metabolism; (6) overexpression of transporters; (7) changes in kDNA; (8) reduced mitochondrial membrane potential; (9) heat shock protein HSP83 and the small kinetoplastid calpain‐related protein (SKCRP14.1) may protect against drug‐induced cell death.

Pentavalent antimonials (Sb[V]) such as meglumine antimoniate (Glucantime) and sodium stibogluconate (Pentostam), are the first‐line drugs used for the treatment of VL, CL, and MCL [76, 77]. These drugs are still in use in East Africa [78]. However, in India, they have been discontinued and replaced with AmB due to issues of drug failure, resistance, and disease relapse [79]. Meglumine antimoniate and sodium stibogluconate are organic complexes of Sb(V) in which the former is prepared by the reaction of Sb(V) and N‐methyl‐D‐glucamine, producing an alternating antimony and N‐methyl‐D‐glucamine complex, whereas the latter is a product of a reaction between Sb(V) and gluconic acid [80, 81]. The drugs are administered intravenously, intramuscularly, or directly into the lesions [82]. Though the mechanism of action is poorly understood, three models have been proposed. The first model is the prodrug model where the Sb(V) are reduced to the toxic and active Sb(III) in the parasite (Figure 4; [83, 84]). It is suggested that the reducing activity is apparent in the amastigotes which is also facilitated by trypanothione under mildly acidic conditions [85, 86]. It is hypothesized that trypanothione forms a complex with Sb(III) which is sequestered via the *Leishmania* ABC transporter, P‐glycoprotein A (PGPA) [87]. It is further suggested that a certain amount of antimonial reduction occurs within the host cells (macrophages), and the Sb(III) enters the amastigotes via the aquaporin 1 (AQP1) transporter. The Sb(V) may also enter the amastigotes through an unidentified transporter to be further reduced to the Sb(III) form [73]. The second model is the active Sb(V) mode where the unreduced form Sb(V) rather than the reduced form Sb(III) exhibits intrinsic antileishmanial activity. The third model is the host immune activation model where the antimonials are able to activate the host immune system (both innate and adaptive) to elicit their antileishmanial activity [77, 88]. Altogether, the antimonials affect various targets including the parasite’s thiol‐redox potential [89], inhibit DNA topoisomerase I [90], inhibit the parasite’s glycolysis and beta‐oxidation in amastigotes [91–93], and induce mitochondrial dysfunction in promastigotes [94].

Improper use and the lack of efficacy are some of the drivers of resistance to pentavalent antimonials [95, 96]. Various mechanisms have been suggested to elucidate the resistance of *Leishmania* to antimonials. Since the susceptibility of the parasite is dependent on the degree of drug reduction, a decreased reduction of Sb(V) to Sb(III) may be a contributing factor of drug resistance. This is supported by a study by Shaked‐Mishan et al. [85] where they observed that resistant amastigote parasites exhibited little or no Sb(V) reducing activity as compared to the wild type. Another mechanism of resistance is the increased production of the rate‐limiting enzymes, which leads to an increase in trypanothione production and thus elevated levels of trypanothione (which aids in the reduction of antimonials) in the parasite [97, 98]. In addition, increased efflux of Sb(III)–thiol complex also promotes resistance [99]. Resistance to pentavalent antimonials also includes the diminished accumulation of Sb(III) in the parasite. This is due to a decreased uptake through the AQP1 transporter, which could be attributed to a gene deletion or a down‐regulation of the transporter [100–102]. Also, the overexpression of the ABC transporter–multidrug‐resistance protein A (MRPA) confers drug resistance both in clinical and laboratory strains, which is associated with elevated levels of trypanothione [103, 104].

### 5.2. Arsenical Derivatives

Melarsoprol (Mel B) is a trivalent melaminophenyl arsenical compound used in the treatment of late‐stage HAT [105]. During melarsoprol uptake in the body, the drug is converted to its active metabolite, MelOx [106]. Melarsoprol/MelOx are taken up by *T. brucei* via the P2 transporter and aquaglyceroporin AQP2 [107–109]. Melarsoprol is also argued to cross the cell membrane via simple diffusion while its metabolite’s uptake is via a transporter [110]. Upon entry into the parasite, MelOx forms a stable adduct with the parasite’s trypanothione (Mel T) [111]. This toxic adduct has also been shown to inhibit the trypanothione disulfide reductase activity in the parasite [111], thus disrupting the redox balance of the cell and its defense against reactive oxygen species leading to death [112]. Melarsoprol also interferes with glycolysis through the inhibition of pyruvate kinase, phosphofructokinase, and fructose‐2,6‐bisphosphatase [113]. The drug also causes rapid loss in cell motility and cell lysis [113]. Alternate transporters for melarsoprol uptake are the high‐affinity pentamidine transporter (HAPT1) and low‐affinity pentamidine transporter (LAPT1) [114], while the efflux of Mel T is mediated via the ABC transporter, MRPA [109].

The functional loss of the P2 transporter activity has been implicated in melarsoprol resistance where the deletion of both alleles of the TbAT1 gene (which encodes the P2 adenosine transporter) stopped the activity of P2, leading to a loss in sensitivity to melarsoprol [114]. A study by Bridges et al. [115] showed that the dual loss of TbAT1 and HAPT1 leads to high levels of arsenical resistance and higher resistance to MelOx. Shahi et al. [116] also demonstrated the overexpression of *T. brucei* MRPA, leading to a 10‐fold increase in resistance to melarsoprol. These observations indicate that resistance mechanisms are linked to reduced drug uptake and increased drug expulsion.

### 5.3. Antibiotics

AmB deoxycholate has been used as a second‐line treatment for VL and MCL especially in HIV patients [117]. However, in the case of antimonial failure, this drug becomes the first‐line treatment [118]. It has been reported that treatment with AmB leads to undesirable side effects such as toxicity, thus the need for a slow but prolonged intravenous administration [118, 119]. To address these issues, liposomal AmB was developed, which has been shown to have a similar efficacy, reduced toxicity, improved half‐life, and ease of administration [120, 121]. AmB acts by binding to ergosterol in the membrane of the parasite, leading to the generation of membrane pores [122]. This creates an efflux of ion and small molecules and the subsequent influx of protons leading to cell death [123, 124]. In addition, AmB induces the production of reactive oxygen species which leads to cell death [125, 126].

Resistance mechanisms of *Leishmania* to AmB include changes in the parasite membrane fluidity. The resistant parasites exhibit enhanced membrane fluidity compared to the wild type, which is attributed to the presence of cholesta‐5, 7, 24‐trien‐3β‐ol in the membrane instead of ergosterol [127]. Reduced levels of thiols and reactive oxygen species are additional characteristics of AmB resistance cells as a result of an overexpression of tryparedoxin peroxidase which could act as antioxidants [128, 129]. This is supported by the fact that AmB resistant clinical isolate showed an increased expression of genes involved in this pathway [130]. In terms of gene expression, particularly in ABC transporters such as the multidrug resistance protein 1 (MDR1) which plays a role in drug efflux, it has been observed that the mRNA levels of this gene were three times higher in resistant clinical isolates compared to the wild type [129]. This further suggests a greater efflux of the drug in the resistant parasite.

Paromomycin is an aminoglycoside antibiotic which is used for the treatment of both CL and VL [118]. The uptake of the drug involves the binding to the cell surface, interacting with surface components, and the internalization via endocytosis [131]. In the parasite, paromomycin binds to the A‐site of parasite’s ribosomes, alters the codon–anticodon recognition process, and induces misreading and inaccurate protein synthesis [132, 133]. Similar to miltefosine, paromomycin also causes mitochondrial dysfunction in the parasite by disrupting the membrane potential and inhibiting mitochondrial dehydrogenase (which affects cellular respiration) [134].

While mutations in the small subunit ribosomal RNA gene of paromomycin‐resistant cells have not been linked to resistance [99, 135], proteomic analysis of paromomycin‐resistant strains has revealed the upregulation of proteins involved in translation, stress responses, metabolism, cell survival, and cell death [136]. These include ribosomal proteins, heat shock proteins, proteins involved in glycolysis, lysophosphatidylglycerol synthesis, and vesicular trafficking [136]. Conversely, signal transduction proteins such as prohibitin were downregulated [136].

### 5.4. Diamidines

Pentamidine is used for the treatment of CL and MCL and the second‐line treatment for VL. However, its use against VL was discontinued due to toxicity and treatment failure [99, 137]. It is also used for the treatment of the first (hemolymphatic) stage of *T. gambiense* HAT [138]. Just like melarsoprol, pentamidine is taken up in trypanosomes via the adenosine‐sensitive pentamidine transporter (ASPT1), which is similar to the P2 transporter, HAPT1 and LAPT1 transporters, and AQP transporters [139, 140], while in *Leishmania*, the drug is taken up via the HAPT1 [141, 142]. Diminazene aceturate is sold under the trade name Berenil and was introduced for the treatment of babesiosis and animal trypanosomiasis in 1995 and has since been the preferred drug for the treatment of animal trypanosomiasis till date. Diminazene uptake in trypanosomes is also mediated by the TbAT1/P2 transporter [143]. Pentamidine and diminazene act by binding to the minor groove of the kDNA, causing a loss in kDNA and interfering with replication and transcription [144–147]. Both drugs also act by accumulating in the mitochondrion, thereby disrupting mitochondrial membrane potential and inhibiting mitochondrial topoisomerase [148–151].

The mechanism of pentamidine resistance involves the reduced uptake of the drug due to loss of transporters, reduced uptake/accumulation of the drug in the mitochondrion, increased efflux of the drug, and alterations in the kDNA sequence [130, 144, 152]. With regards to transporters, it was demonstrated that loss in the TbAT1/P2 confers diminazene resistance to trypanosomes [143]. In *T. congolense*, resistance to diminazene is associated with reduced mitochondrial membrane potential [153]. A study by Basselin et al. [141] showed that pentamidine resistance in *Leishmania* is characterized by reduced/slow accumulation of the drug in the mitochondrion and also the efflux of the drug from the plasma membrane of the cell. This resistance mechanism is supported further through biochemical analysis where the use of mitochondrion metabolic inhibitors reduced the uptake of drug, thereby emphasizing its importance as a mechanism of resistance [130]. Also with regards to efflux, the pentamidine resistance protein 1 (PrP1) has been shown to be involved in resistance. This ABC transporter is suggested to act as an efflux pump which eventually reduces the accumulation of the drug in the mitochondrion [130, 154, 155]. At the molecular level, resistance to pentamidine could also be due to changes in the sequence of the minicircle of kDNA, which results in fewer binding sites for the drug [144]. Cross resistance between melarsoprol and pentamidine has also been reported, where, through the use of methods including genome‐scale RNA interference target sequencing (RIT‐seq), loss and mutations of the AQP transporter genes (i.e. loss of AQP2 and chimerization of AQP2–AQP3) have been implicated [70, 151, 156]. More recently the CRISPR‐based diagnostic‐specific high‐sensitivity enzymatic reporter unlocking (SHERLOCK) has also been used to detect known mutations in the aquaporin transporters, which have been associated with melarsoprol and pentamidine cross resistance in trypanosomes [157].

### 5.5. Phospholipid Analogs

Miltefosine is the first oral drug for the treatment of VL with a curative rate of 98% [118, 158]. The uptake of miltefosine by the parasite is facilitated by the miltefosine transporter–Ros3 complex (MT–Ros3), which is also responsible for the transport of phospholipids [159]. Once in the parasite, the drug acts by interfering with the composition and fluidity of the cell membrane and membrane lipids by inhibiting the biosynthesis of phosphatidylcholine, sterol, and fatty acid [160, 161] and inducing mitochondrial dysfunction by reducing the mitochondrial depolarization and the activity of cytochrome c [162]. The drug further acts by activating the parasite’s calcium channel, causing disruption of the its calcium homeostasis and impairing acidocalcisomes activity [163]. Miltefosine also induces apoptosis‐like cell death which is characterized by DNA condensation and fragmentation [164]. The immunomodulatory effect of miltefosine involved the drug induction of interleukin‐2 production in T cell hybridoma and an increase in the production of IFN‐γ, interleukin‐12, and TNF‐α in the macrophages [165, 166]. Miltefosine has also been shown to inhibit mitosis [147].

Resistance to miltefosine is generally related to a reduction in drug uptake. A study by Pérez‐Victoria et al. [167] showed that a two single‐point mutation in the miltefosine transporter gene causes a loss of function in the transporter, which eventually impairs the uptake of the drug. Likewise, point mutations or gene deletions in the Ros3 gene also obstruct the uptake of miltefosine by the parasite [159]. The overexpression of genes of the ABC transporters such as the P‐glycoprotein–like transporter and ABCG‐like half‐transporters confers significant resistance to miltefosine which is also characterized by a reduced drug uptake [168–170]. Other resistance mechanisms include the protection against miltefosine‐induced cell death, which is facilitated by proteins such as heat shock protein HSP83 and the small kinetoplastid calpain‐related protein (SKCRP14.1) (Figure 4; [171]).

### 5.6. Nitroheterocyclic Derivatives

Nifurtimox, benznidazole, and fexinidazole are oral drugs used in the treatment of kinetoplastid diseases. Nifurtimox and benznidazole are used in the treatment of American trypanosomiasis (Chagas disease) caused by *T. cruzi* [172, 173]. Nifurtimox is also used for the late‐stage treatment of *T. gambiense* HAT, especially in melarsoprol refractory cases [174], while fexinidazole is used in the treatment of the early and last stage of *T. gambiense* HAT. The development of fexinidazole monotherapy in the treatment of *T. cruzi* has been discontinued due to low efficacy despite a satisfactory safety profile [175]. All three drugs act as prodrugs; thus, these drugs must be activated to elicit their cytotoxic effect which is aided by the Type I NTRs [176–178]. Fexinidazole is also metabolized by the host into sulfoxide and sulfone derivatives which have similar activity as the unmetabolized form [179, 180]. Nitroheterocyclic compounds cause cellular damage to cells through the generation of free radicals and/or electrophilic intermediates [106, 181], though Boiani et al. [182] have argued otherwise. A study by Thomas et al. [147] indicated that nifurtimox causes a loss in mitochondrial membrane potential, affecting mitochondrial protein import and thus a reduction in the protein abundance.

Resistance to these medications has been linked to the activity of Type 1 NTR. A loss or mutation in NTR (as seen with nifurtimox and benznidazole) [177, 183] or the deletion of its transcriptional unit (as in the case of fexinidazole) [72] results in decreased levels of NTR expression, thereby leading to resistance. Cross resistance to these drugs has also been reported [177, 179].

### 5.7. Amino Acid Derivatives

Eflornithine (DL‐α‐difluoromethylornithine [DFMO]) is an amino acid ornithine analog used in the treatment of late‐stage *T. gambiense* HAT and in melarsoprol refractory cases [184]. Uptake of DFMO is via the amino acid transporter gene, TbAAT6, which belongs to the amino acid transporter 1 superfamily [156, 185, 186]. DFMO is an irreversible inhibitor of ornithine decarboxylase (ODC), a key enzyme in the synthesis of polyamines, spermidine, and spermine, which are important for cell proliferation [187, 188]. The drug depletes the parasite’s polyamines and spermidine and also causes a reduction in the levels of trypanothione, leading to the morphological change of the parasite from the long slender to the short stumpy form, which are incapable of cell division/binary fission [113, 189]. Resistance to DFMO is associated with the TbAAT6 transporter where gene knockdown and deletion lead to reduced drug uptake, thus conferring resistance [156, 185, 186].

### 5.8. Urea Derivatives

Suramin is used for the treatment of early‐stage *T. rhodesiense* HAT and for *T. evansi* in camels and horses. The uptake of suramin by trypanosomes occurs through the receptor‐mediated endocytosis route which involves the pathway invariant surface glycoprotein ISG75 and the subsequent transportation to the cytosol facilitated by the lysosomal transporter [156, 190]. In the parasite, suramin reduces the cellular content of the cell and glycolytic ATP production through the inhibition of glycolytic enzymes in the cytosol [113, 191], which eventually leads to cell death. Suramin is also suggested to interfere with DNA synthesis by inhibiting the activities of dihydrofolate reductase and thymidine kinase [113, 192, 193]. As with most drugs, the reduced uptake of suramin confers resistance, which was demonstrated through RNAi‐mediated knockdown of ISG75 [156].

### 5.9. Phenanthridine

Isometamidium chloride is used in the treatment of AAT and is effective against *T. congolense* and other trypanosome species [194]. In *T. congolense*, uptake of isometamidium chloride is by a receptor‐mediated transport system found on the surface of the parasite [195]. This transport system has also been shown to be energy‐dependent [195], while in *T. brucei*, some amount of the drug is taken up via the P2 transporter [196]. In the parasite, the drug binds to kDNA [197], accumulates in the mitochondrion, and inhibits the mitochondrial Type II topoisomerase, resulting in impaired mitochondrial DNA structure, loss of kDNA, and cell death [146].

Resistance to isometamidium involves reduced accumulation of the drug [198], drug efflux due to changes, replacement or mutations of the transporters [195, 199], and disrupted mitochondrial membrane potential resulting in decreased drug accumulation [200]. Cross resistance to diminazene has also been reported [201, 202]. Table 1 and Figure 4 summarize the chemical structures and resistance mechanisms of major antitrypanosomatid drugs.

**TABLE 1:** Antitrypanosomatid drugs and possible mechanisms of resistance.

Drug	Structure	Mechanism of resistance	Reference
Pentavalent antimonials: meglumine antimoniate (Glucantime) and sodium stibogluconate (Pentostam)	 Meglumine antimoniate (Glucantime)	Decreased reduction of SbV (inactive) to SbIII (active and toxic); Increase in trypanothione production; increased efflux of Sb(III)–thiol complex; gene deletion or a downregulation of the AQP1 transporter; overexpression of The ABC transporter–multidrug‐resistance protein A (MRPA)	[85, 97–104]
	 Sodium stibogluconate (Pentostam)		

Melarsoprol		Functional loss of the P2 transporter activity due to deletion of both alleles of the TbAT1 gene; dual loss of TbAT1 and HAPT1 transporters; overexpression of *T. brucei* MRPA	[70, 114–116]
		Loss of AQP2 and AQP2–AQP3 chimerization	

Amphotericin B and liposomal amphotericin B		Changes in the parasites’ membrane fluidity; overexpression of tryparedoxin peroxidase; increase in mRNA levels of ABC transporters like the multidrug resistance protein 1 (MDR1) which plays a role in drug efflux	[127–130].

Paromomycin		Upregulation of proteins involved in translation, stress responses, metabolism, cell survival, and cell death	[136].

Pentamidine		Reduced uptake of the drug due to loss of transporters, reduced/lack of uptake/accumulation the drug in the mitochondrion, increased efflux of the drug, and alterations in the kDNA sequence; pentamidine resistance protein 1 (PrP1) involved in efflux; loss of AQP2 and AQP2–AQP3 chimerization	[70, 130, 141, 144, 152, 154, 155].

Diminazene		Loss in the TbAT1/P2 transporter; reduced mitochondrial membrane potential	[143, 153].

Miltefosine		Single‐point mutation in the miltefosine transporter gene causes a loss of function in the transporter; point mutations or gene deletions in the Ros3 gene; overexpression of P‐glycoprotein–like transporter and ABCG‐like half‐transporters; heat shock protein HSP83 and the small kinetoplastid calpain‐related protein (SKCRP14.1) protect against miltefosine‐induced cell death	[159, 167–171].

Nifurtimox and benznidazole	 Nifurtimox	A loss or mutation in NTR	[177]
	 Benznidazole		

Fexinidazole	 Fexinidazole	Deletion NTR transcriptional unit	[72]

Eflornithine		Gene knockdown and deletion of TbAAT6 transporter	[156, 185, 186]

Suramin		Reduced uptake of suramin due to low expression of ISG75	[156]

Isometamidium chloride		Changes, replacement, or mutations of the transporters; disrupted mitochondrial membrane potential	[195, 199, 200]

Pafuramidine/furamidine		Loss of P2‐mediated uptake	[203]

Benzoxaborole: acoziborole		Single‐nucleotide polymorphism in multiple genes including CPSF3	[204]

LXE 408		Mutation in β4 subunit of the proteasome	[205]

### 5.10. Other Drugs/Compounds

Pafuramidine (DB289) is a prodrug of furamidine (DB75), which is a diamidine analog of pentamidine [206]. This orally administered drug is biotransformed in the liver to its active metabolite, furamidine. Pafuramidine was used to treat the first stage of HAT during a Phase III clinical trial and showed comparable efficacy to pentamidine [207]. Once pafuramidine has been converted the furamidine, the latter is taken up by the parasite via the P2 aminopurine transporter (TbAT1) [203]. The mode of action of pafuramidine is associated with furamidine and similar to diamidine; furamidine also accumulates in the nucleus, kinetoplast, binding to the minor groove DNA [208, 209]. It also accumulates in the acidocalcisomes and mitochondrion of trypanosomes [208, 210]. This accumulation may potentially affect cellular processes such as DNA replication and disrupt the mitochondrion membrane potential [210, 211]. A loss of the transporter‐mediated uptake of the drug confers resistance [203]. The development of pafuramidine as a potential antitrypanosomal drug was discontinued due to toxicity issues, some of which affected the liver and kidney [206, 207].

Benzoxaboroles are a class of compounds showing strong potential as therapeutic agents for the treatment of HAT [212, 213]. Acoziborole (SCYX‐7158), which belong to the benzoxaborole class, has advanced to the Phase II/III clinical trials for the treatment of gambiense‐HAT [214]. This orally administered drug stands out as a promising therapeutic candidate owing to its potent efficacy and good safety profile [214]. In trypanosomes, acoziborole targets the parasite’s cleavage and polyadenylation specificity factor 3 (CPSF3), which is the nuclear mRNA processing endonuclease, thus inhibiting mRNA maturation and processing [215, 216]. The mechanism of resistance is not straightforward as genomic studies of resistant cell lines identified single‐nucleotide polymorphisms in multiple genes including CPSF3 [204]. Similar to acoziborole, AN2‐502998, which belongs to the benzoxaborole class of compounds, is an orally active inhibitor of the CPSF3 inhibitor in *T. cruzi*, positioning it as a potential treatment for chronic Chagas disease [217]. This drug is currently advancing toward Phase I clinical trials [217].

LXE408 is a selective proteasome inhibitor and exhibits good antitrypanosomatid activity [218]. This oral drug is currently used in a Phase II clinical trial for the treatment of VL in India and Ethiopia [218]. The drug selectively targets the proteasome of *Leishmania,* acting as noncompetitive inhibitor of the parasite’s chymotrypsin‐like activity [205]. A mutation in the β4 subunit of the proteasome confers selective resistance to LXE408 [205].

Recent studies have focused on exploring the GSK Kinetobox to identify novel inhibitors targeting pteridine reductase 1 (PTR1) and dihydrofolate reductase–thymidylate synthase (DHFR–TS) [219]. These enzymes play key roles in the folate metabolic pathway and represent attractive therapeutic targets for the treatment of trypanosomatid infections [220]. One of such inhibitor, TCMDC‐143249, a benzenesulfonamide derivative, has demonstrated selective inhibition of PTR1, effective growth inhibition, and in vivo efficacy against *Trypanosoma* and *Leishmania* species [219]. Thus, TCMDC‐143249 could serve as a promising candidate for further drug development.

Emerging evidence indicates that genomic plasticity plays a central role in drug resistance, particularly in *Leishmania* species. Unlike most eukaryotes, *Leishmania* exhibits mosaic aneuploidy and extensive gene copy number variation, enabling rapid adaptation to environmental stress, including drug pressure [221, 222]. Amplification or deletion of chromosomes and gene clusters can modulate drug targets, transporters, and metabolic pathways, thereby conferring resistance.

In addition to genetic changes, the presence of “persister” parasite populations has been proposed as a mechanism underlying treatment failure and relapse. These subpopulations exhibit transient drug tolerance without stable genetic resistance and can survive prolonged drug exposure, later repopulating the infection once treatment ceases [223, 224]. This phenomenon parallels bacterial persistence and represents a significant challenge for achieving complete parasite clearance.

Metabolic rewiring has also been recognized as a key adaptive response to drug pressure. Trypanosomatids can alter mitochondrial function, lipid metabolism, and redox balance to survive exposure to chemotherapeutic agents. For example, enhanced antioxidant defenses and shifts in energy metabolism have been linked to resistance against AmB and antimonials. These findings emphasize that drug resistance is a multifactorial process involving coordinated metabolic and regulatory changes rather than single‐gene mutations alone.

## 6. Management and Control of Drug Resistance

Although significant progress has been made in the development of drugs for the control of these parasites, the emergence of drug resistance threatens the efficacy of current therapies. To effectively combat drug resistance in trypanosomiasis and leishmaniasis, it is crucial to understand the mechanisms underlying resistance development. One approach that has been used to combat drug resistance is the use of combination drugs (Table 2). Different drugs with distinct mechanisms of action may reduce the likelihood of resistance development when combined. This suggests that the efficiency of combining drugs may be based on the synergistic effects of the drugs. In animal trypanosomiasis, a combination of the trypanocidal drugs diminazene, homidium, and isometamidium has been explored. According to a study by Dehoux [225], a combination of diminazene aceturate and isometamidium chloride was used in Zaire to protect cattle from trypanosomiasis. Again, standard doses of either diminazene aceturate or isometamidium chloride were not able to clear resistant strains of *T. evansi*, whereas their combination lowered the parasitemia drastically [226]. The combination of diminazene aceturate and isometamidium chloride has also been found to exhibit a higher efficacy against *T. brucei*, *T. congolense*, and *T. vivax* compared to the single drugs in a high tsetse fly density area [227]. A study by Ihedioha et al. [228] also showed that the coadministration of diminazene aceturate and Na‐EDTA in diminazene‐resistant *T. brucei* resulted in low parasitemia density, improvement in packed cell volume, and postinfection survival time. In a comparative study of homidium and diminazene, it was again observed that the combination therapy reduced parasitemia significantly compared to the single therapy. It was thus concluded that due to the higher efficacy of the combination therapy, it could be used as a temporary measure to mitigate the effect of drug resistance [229]. Another study also suggested that Cymelarsan and suramin do not induce cross‐reactivity to each other experimentally; hence, both drugs can be combined for the treatment of *T. evansi* [230, 231].

**TABLE 2:** Common management and control strategies for trypanosomatids.

Strategy	Approach	Role in resistance control	Example	Reference
Combination therapy	Use of 2 or more drugs with different modes of action	Reduction in selection of resistant strains	Diminazene aceturate and isometamidium chloride	[226]
			Nifurtimox and eflornithine	[232]
			Paromomycin, miltefosine, and amphotericin B	[233]

New drugs	Development of novel drugs	Overcome existing resistance	Simeprevir	[234].
			5‐Nitroimidazole	[235]

Rational drug usage	Avoid counterfeit drugs	Reduces selective pressure of drugs	Trypanosomatid drugs	[236]
	Correct dosage, etc.			

Vector control	Reduce transmission	Prevents spread of resistant strains	Insecticide‐treated targets, spraying and use of traps	[237]

Surveillance	Monitor resistance patterns	Early detection	Molecular markers	[238]

Vaccination	Development of vaccines	Reduce over reliance on drugs	ISG75	[239, 240]
			Invariant surface proteins	

Targeting resistant genes	Identify potential drug‐resistant marker	Restores drug sensitivity	Transporter inhibitors	[241, 242].

Molecular diagnostics	Early detection	Ensures correct treatment	PCR, PCR–RFLP, etc.	[243]

Public health/capacity building	Improve adherence	Proper drug usage	Awareness programmes	[244].
		Reduces drug misuse		

In human trypanosomiasis, the combination of drugs has also been shown to be more effective than the single ones. For example, the combination of nifurtimox and eflornithine has shown increased efficacy compared to monotherapy, providing a more robust treatment strategy [232]. The same combination has been shown to be effective against the second‐stage *T. brucei gambiense* in humans [245]. Nifurtimox has been reported to have a synergistic effect with melarsoprol, and their combination was shown be more effective in the treatment of the second‐stage *T. brucei gambiense* trypanosomiasis with little or no relapse compared to treatments due to monotherapies [235, 246]. For human trypanosomiasis, although several combinations of drugs have been tried, the nifurtimox–eflornithine combination has been shown to be superior in terms of effectiveness and safety, thus has been used as the first‐line treatment drug [232, 235, 245].

However, there are reports of trypanosomes having multiple resistance to trypanocidal drugs including the combination therapies, hence questioning their effectiveness [247, 248]. It has also been shown that selection of resistance to eflornithine in the laboratory is relatively easy (due to loss of an amino transporter involved in drug uptake), as it is for nifurtimox [249]. In leishmaniasis, the combination of drugs such as paromomycin, miltefosine, and AmB has been shown to be effective and safer in the treatment of the disease compared to the monotherapies [233, 250, 251]. These combinations do not only improve the efficiency of treatment but also crucial in preventing drug resistance. A study in India showed that the combination of liposomal AmB (Fungisome) and miltefosine improved clinical parameters and efficacy and reduced toxicity in individuals with VL compared to the monotherapy drug miltefosine [233]. In another study, it was shown that combining liposomal amphotericin with miltefosine achieved cure rates above 95% with fewer relapses compared to either drug alone [250].

Another factor that could mitigate the effect of drug resistance in trypanosomatids is the rational use of drugs. Appropriate and rational use of antiparasitic drugs is crucial in minimizing the development of drug resistance. This includes prescribing drugs only when necessary, adhering to recommended dosages and treatment protocols, preventing the use of substandard or counterfeit drugs, and ensuring that drugs are only given when they are clinically needed [252]. Effective drug management helps maintain drug efficacy and reduces the selective pressure on trypanosomes to develop resistance [237]. Studies have reported that some of the major risk factors for trypanocidal resistance are acquisition of trypanosomal drugs from unofficial sources, frequent treatment regimens, treatment of infected animals by untrained personnel, and noncompliance to quality drugs [236, 253]. It was suggested that inadequate veterinary services and high costs of the drugs from authorized persons were responsible for the farmers acquiring the drugs from illegal sources [236]. In another study, it was reported that exposure of parasites to subtherapeutic drug doses as a result of underdosing, uncontrolled used of antitrypanocidal drugs, and lack of proper diagnosis of the disease may increase the chance of development of drug resistance [254]. According to Clausen et al. [252], information and training of farmers on rational drug usage improved farming practices and reduced underdosage issues and thus significantly prevented drug resistance. Again, it also helped the farmers in the areas of vector‐control practices, knowledge on the disease and nutrition, diagnosis, and treatment of the disease. It was thus suggested that underdosage may be due to the fact that most farmers underestimate the weight of their animals hence not giving them the right dose.

Another way to contain the development of resistance is the development of new therapies [255]. A major challenge of this strategy is the cost implication. Even in the face of this challenge, new drug candidates are currently being developed. Currently, 5‐nitroimidazole (analog of fexinidazole) has been successfully characterized and shown to be effective against the treatment of both *T. b. gambiense* and *T. b. rhodesiense* [180, 235, 256]. Another compound oxaborole SYX‐7158 (benzoxaborole) which was developed against Stage II HAT was shown to effectively clear parasites in mice models [235, 257]. A distinct derivative of benzoxaborole similar to that used in the treatment of HAT was also developed to prevent and treat AAT [258]. Unlike mammals, protozoan parasites are unable to synthesize essential purine nucleosides and hence use enzymes from the purine salvage pathway to acquire purines from the host [259, 260]. This suggests that analogs of purine nucleosides may act as potential antitrypanosomal agents. The strategy has been used to propose potential antitrypanosomal drug candidates such as tubercidin [261], cordycepin [262], and formycin [263]. For leishmaniasis, it has been reported that through new approaches such as drug repurposing, nanotechnology, and host‐directed therapy, new compounds with potential antileishmanial activity have been identified [264]. Some of the compounds include sertraline, simeprevir, rapamycin, leptin, oleuropein, and simvastatin. Simeprevir, an antiviral drug, has been shown to be an inhibitor of sterol C‐24 methyltransferases of *L. donovani*. It inhibits *L. donovani* growth of promastigotes with 50% IC_50._ [234]. Through drug repurposing, another drug sertraline has been shown to significantly decrease intracellular ATP levels and oxidative stress in *L. infantum* promastigotes. It causes a remarkable variation of the levels of thiol‐redox and polyamine biosynthetic intermediates, as well as a shortage of intracellular amino acids used as metabolic fuel by *Leishmania*. It therefore suggests that sertraline killed *Leishmania* through a multitarget mechanism of action, tackling essential metabolic pathways of the parasite [265]. Rapamycin has been shown to be an alternative drug against leishmaniasis by reducing the parasite load in BALB/c mice infected with *L. tropica* [266]. Leptin has been found to affect M1 macrophage polarization in *L. tropica* infection by enhancing the efficacy of glucantime. This is achieved by its ability to increase the expression of M1‐associated markers (CD86, iNOS, SOCS3, and miR‐155) and proinflammatory cytokines (TNF‐α, IL‐12, and IFN‐γ) while decreasing M2‐associated markers (CD206, ARG1, SOCS1, and miR‐146a) and anti‐inflammatory cytokines (IL‐4, IL‐10, and TGF‐β) [267]. For oleuropein, it was found to act as an immunomodulatory agent by promoting a Th1‐type immune response in *L. donovani*–infected BALB/c mice. Results showed that it was able to mount a significant Th1 polarization characterized by the expression of immune genes (IL‐12β, IL‐10, TGF‐β1, and IFN‐γ) and transcription factors (Tbx21 and GATA3) [268]. Finally, simvastatin was shown to enhance host protection against *L. major* by increasing macrophage phagosome maturation and killing effector functions [269].

Understanding the genetic and molecular mechanisms underlying drug resistance in trypanosomiasis and leishmaniasis is crucial for effective management and control. Genetic studies can identify resistance markers and mechanisms, enabling the development of diagnostic tools to detect drug‐resistant strains. These studies can provide insights into the molecular targets of drugs and help optimize drug design. These approaches aided in the identification of the gene encoding the P2 adenosine transporter in trypanosomes [108] and the amino acid transporter AAT6 [185], which are responsible for the uptake of the drugs melarsoprol and eflornithine, respectively, in *T. brucei*. A reversed genetics‐based approach has also been used to identify potential drug resistance markers melarsoprol–trypanothione (Mel‐T) transporter, MRPA [116], and the transporters NT11‐1 and NT12‐1 responsible for uptake of pentamidine [241]. Furthermore, Alsford et al. [270] used high‐throughput genetic screening approaches to identify potential resistant genes of HAT drugs. Similar genetics approaches have also been used to identify drug‐resistant markers in leishmaniasis. Some of the resistance genes identified include aquaglyceroporin 1 (AQP 1), ABC transporters, protein 299 (P299), amino acid permease, ubiquitin, etc [242]. The identification of these drug resistant genes has aided in the understanding of the mechanisms responsible for drug resistance in kinetoplastids. The mechanisms have thus helped in the control and management of drug resistance and development of new drugs to prevent drug resistance.

The main vectors responsible for trypanosomiasis and leishmaniasis are tsetse flies and sand flies, respectively. Implementing vector control measures, such as insecticide‐treated targets, insecticide spraying, and the use of traps, can help reduce the transmission of the parasites responsible for these diseases and subsequently decrease the reliance on drug treatment. Vector control efforts contribute to minimizing the risk of drug resistance development in trypanosomes [237, 271]. According to Matovu et al. [272], the involvement of vectors in drug resistance is due to the fact that there are specific genes which are involved in the regulation of drug resistance. A study suggested that there is genetic exchange between different trypanosome species within the vector; thus, the evolution of resistance is dependent on the extent of genetic exchange in the vector and transmission intensity [273]. A recent study in Cameroon used the polymerase chain reaction–restriction fragment length polymorphism (PCR–RFLP) to identify drug‐resistant trypanosomes in tsetse flies [243]. Results showed that the study site had a high vector density especially tsetse flies, and there was the existence of diminazene aceturate–resistant strains of *T. congolense* in the tsetse flies. It was thus concluded that the detection of the drug‐resistant trypanosomes in the tsetse flies will aid in mapping of specific areas where trypanosomes are transmitted and also to improve the strategies to control drug resistance. A review by Aksoy et al. [274] reported the development of genetically modified tsetse flies to render resistance to trypanosome infections. The resistant strains are released into the wild so they can compete with the ones which are not modified to suppress and prevent transmission of parasites. The inability of transmitting the parasites may prevent or reduce the development of drug resistance.

Emerging frontiers of research in control strategies present new opportunities for therapeutic interventions. Recent studies have revealed that host–parasite metabolite exchange is far more dynamic and essential to trypanosomatid survival than previously appreciated. Rather than relying solely on intrinsic metabolic pathways, parasites such as *Leishmania* and *Trypanosoma* spp. actively scavenge and remodel host‐derived metabolites, including lipids, amino acids, and nucleotides, to sustain intracellular growth and virulence [275, 276]. Metabolomic and flux analyses have demonstrated that these parasites can reprogram host cell metabolism (particularly macrophage glycolysis and lipid handling) to create a permissive niche for replication. This metabolic interdependence presents new opportunities for therapeutic intervention targeting host–parasite metabolic interfaces.

Another emerging concept is parasite quorum sensing, particularly well characterized in *T. brucei.* Parasites release and respond to density‐dependent signaling molecules that regulate differentiation from proliferative slender forms to nondividing stumpy forms, which are preadapted for transmission to the tsetse fly [277]. This process ensures optimal parasite survival and transmission efficiency while preventing premature host death. The identification of signaling pathways and molecular mediators involved in quorum sensing has opened new avenues for disrupting parasite development and transmission cycles.

Hybridization and genetic recombination have also gained attention as drivers of diversity and adaptation in trypanosomatids. Evidence of genetic exchange in *T. brucei* within the tsetse fly vector and in *Leishmania* spp. has challenged the long‐held view of strictly clonal reproduction [278, 279]. Hybrid strains may exhibit altered virulence, host range, and drug susceptibility, raising concerns about the emergence of more resilient parasite populations. Advances in whole‐genome sequencing have facilitated the detection of recombination events and introgression, highlighting their epidemiological and clinical significance.

The impact of climate change on the distribution and transmission of trypanosomatid diseases is an increasingly important area of research. Changes in temperature, rainfall patterns, and land use are influencing the geographic range of vectors such as tsetse flies and sand flies, potentially expanding disease endemicity into previously unaffected regions [280]. Predictive ecological models integrating climate data with vector biology are being used to forecast future transmission hotspots and guide public health interventions. This underscores the need for adaptive surveillance systems that can respond to shifting epidemiological landscapes.

Other factors such as surveillance and monitoring, vaccination, global collaboration, public health education, and capacity building are also worth considering. Regular surveillance and monitoring of drug resistance patterns and prevalence are crucial for early detection of the emergence of drug resistance. Early monitoring of the prevalence of drug resistance in kinetoplastids has been suggested to aid in treatment strategies, implementation of proper control measures, prevention of the spread of drug‐resistant strains, and identification of hotspot areas of drug‐resistant parasites [238, 281]. Vaccine development has been considered as one of the surest alternative approaches to control trypanosomiasis and leishmaniasis and reduce the over reliance on drugs. Several potential vaccine candidates targeting different stages of these diseases have been identified [239, 240, 282, 283]. Engagement of communities through awareness creation and educational programs can help to promote the importance of proper drug usage, adherence to treatment protocols, and the consequences of drug resistance. This approach may also be crucial for the success of mass drug administration and sustainable control programs. Apart from this, strengthening of healthcare infrastructure and building capacity in endemic regions are crucial for the control and management of drug resistance. Capacity building may involve training of health professionals, improving diagnostic capacities and research collaborations, and ensuring access to good quality drugs for treatment [244].

## 7. Potential Applications of AI

Machine learning algorithms have evolved to aid in high throughput and increased sensitivity, accuracy, and specificity for parasite detection. Various algorithms have been employed in detecting parasites in samples such as blood smears and stool based on characteristic features such as shape and staining pattern of parasites.

In a study by Rajaraman et al. [284], a machine learning approach based on the random forest (RF) algorithm was coupled with a low‐resolution camera for parasite detection. This cost‐effective method, using mobile phones with low resolution, yielded higher sensitivity, specificity, and precision values of 90.5%, 0.942%, and 87.6%, respectively [284]. Morais et al. [285] conducted a similar study where they utilized the RF algorithm to identify and quantify *T. cruzi* trypomastigotes in blood smears using low‐resolution mobile phones. Despite using low resolution images (less than 1 megapixel), the algorithm obtained a higher sensitivity and specificity of 90.5% and 88.6% respectively, thereby serving as a potential alternative cost‐effective method for AI diagnostic methods for *T. cruzi* trypomastigote [285].

Convolutional neural networks (CNNs) are widely recognized for their remarkable effectiveness in tasks such as image classification and segmentation, significantly advancing the field of deep learning. By leveraging CNNs alongside sophisticated data analysis and machine learning algorithms, researchers have been able to develop predictive models crucial for detecting parasites [286]. The U‐Net system operates by segmenting input images pixel‐wise using a combination of convolutional and deconvolutional networks [287, 288]. This architecture integrates a contracting path for feature extraction with an expanding path for precise localization. In the context of parasite detection, the system was trained using promastigote, amastigote, adhered parasite, nucleus, cytoplasm, background, and other nonparasitic regions were annotated as unknown. These annotations were generated from microscopic images captured from *Leishmania* spp.– infected (*L. infantum, L. braziliensis*, and *L. major*) macrophages, as reported by Górriz et al. [288]. This enables accurate identification of parasitic structures, segmentation, and classification within images by learning from annotated examples. By leveraging deep learning, the U‐Net enhances the efficiency and accuracy of parasite detection in medical imaging, offering automated and reliable diagnostic capabilities [288]. Sanchez‐Patiño et al. [289] achieved an accuracy of 99.19% and a Jaccard index of 49.43% when they trained a U‐Net CNN for diagnosis of *T. cruzi* amastigotes nests, making it efficient for diagnosis of *T. cruzi* in histopathological images of endomyocardium biopsy.

Another AI‐based diagnostic tool that holds significant promise for improving the accuracy and efficiency of diagnosing leishmaniasis is the deep learning algorithm AlexNet, which is a CNN widely recognized for its proficiency in image classification tasks. Through a process known as transfer learning, AlexNet leverages pretrained networks to learn from image datasets, making it particularly effective in image analysis. The AI system is trained using a dataset comprising images of CL lesions alongside other skin diseases. During its training phase, AlexNet undergoes the process of learning to recognize specific patterns and features associated with skin lesions, enabling it to accurately distinguish them from other skin conditions [290, 291]. A study conducted by José et al. demonstrated that AlexNet achieved an impressive accuracy rate of 95% in identifying CL lesions, highlighting its efficacy in diagnosing this condition [291]. MobileNet V2 is another valuable CNN tool. MobileNet V2 analyzes microscopy images of peripheral blood samples spread on tiles and feeds them into a single‐neuron classifier. In a study by Pereira et al. [292], MobileNet V2 convolutional layers were applied to analyze image tiles extracted from acute‐phase peripheral blood samples. The resulting 1280‐dimensional feature vectors were then fed into a single‐neuron classifier with an accuracy of 95.4%. This results can be improved by employing MobileNet V3‐Small, a next generation MobileNet based on a combination of complementary search techniques and architecture design, with image classification of 6.6% more accurate compared to MobileNet V2 [293].

Zare et al. [294] developed an AI system utilizing morphological data from slide assessments. The system is based on the Viola–Jones algorithm bolstered by the adaptive boost (AdaBoost) method. It is designed for detecting infection in collected smears, whether stained or unstained, with a fairly high degree of specificity and a moderate level of sensitivity. This AI system undergoes training using datasets containing both parasitic and nonparasitic samples, facilitating the acquisition of discernible features, enabling the system to effectively detect infected regions.

The Viola–Jones algorithm was used to identify the CL parasite within infected macrophages, achieving a recall rate of 65% and precision of 50%, while the detection of amastigotes outside macrophages yielded a recall of 52% and precision of 71% [294, 295]. Similar but modified techniques for parasite detection have been exploited in detecting other parasites such as Chagas (*T. cruzi*) and malaria parasites in blood smears [296, 297].

The support vector machine (SVM) method, together with AdaBoost, was employed in parasite detection involving four stages: image acquisition, conversion to grayscale format, parasite detection, and postprocessing. In the image acquisition stage, blood smears stained with Wright stain are dried and imaged using microscopy. The image data are simplified by converting into grayscale by combining specific percentages of RGB values and stored in a matrix format. The detection stage utilizes Haar‐like features assisted with AdaBoost binary classifiers to enable the identification of shrimp‐shaped Chagas parasites in the grayscale images. The final stage, postprocessing, involves the use of a postprocessing filter to reduce false positives by analyzing dark DNA spots characteristic of parasites, differentiating them from nonparasitic entities [296].

The mobile bot application devised by Jomtarak et al. for automatic detection of *T. evansi* infections operates based on a deep learning approach, leveraging YOLO neuronal network algorithms to predict *T. evansi* blood stages via thin‐blood film analysis. The system conducts image classification and object detection utilizing bounding boxes. YOLO v4 demonstrates superior performance in multiscale detection, identifying and categorizing novel images with remarkable accuracy metrics, including 95% sensitivity, specificity, precision, accuracy, and F1 score, with a misclassification rate of less than 5%. Its application extends to identifying skin lesions, potentially beneficial for detecting CL [298, 299]. The CiRA CORE platform integrates YOLO v4 for trypanosome detection, employing object identification and classification neural networks to discern and categorize *T. cruzi*, *T. brucei*, and *T. evansi* species from oil‐immersion microscopic images. Utilizing pattern recognition, the system scrutinizes multiple protozoa in a single blood sample, highlighting distinctive features such as the nucleus and kinetoplast using an attention map [300].

Machine learning algorithms can be applied to analyze genomic data to identify specific mutations associated with drug resistance [301]. Genomic data analysis aids in the examination and identification of genomic markers that contribute to drug resistance [302], through the utilization of RFs which are used for classification and regression of genomic data [303]. Predictive modeling has also been another AI tool to detect drug resistance in protozoans. Deep learning models can be used to predict the likelihood of resistance to a particular drug based on the presence of specific mutations. These models can learn from large datasets and identify patterns indicative of drug resistance [304].

The application of machine learning with the Sci‐kit learn library in Python involved using molecular fingerprint vectors as input to classify molecules into active and inactive. Linear regression (LR), gradient boosting (GB), RF, and SVM were the algorithms used to assess accuracy against targets of kinetoplastid proteins. RF and SVM demonstrated high values in this assessment. Receiver operating characteristic (ROC) and precision recall (PR) curves were generated, and their area under the curve was used to evaluate performance, including sensitivity, specificity, precision, balanced accuracy, and F1 score. This classification aided in generating kinetoplastid effectors for approved and future drugs. Protein structures in *Leishmania* spp. for drug targets were obtained from the Protein Data Bank and prepared for docking simulations using the Open Babel software [305]. The AutoDock Vina program has also been used to dock ligands against their receptors [306].

The Ligand Similarity using Clique Algorithm (LiSiCA v1.0) software is used to search for two dimensional (2D) bioactive molecules that can perform inhibitory actions against kinetoplastids. This AI tool utilizes the Tanimoto coefficient, a statistical method used to assess the similarity and diversity of sample sets, to conduct a 2D molecular similarity search among compounds. Similar compounds or those closely related to the bioactive molecule are identified and subjected to molecular docking. Compounds that achieve a docking score within the same range as the reference molecule are further tested in vitro to validate their efficacy against the specific parasite. In a study involving *Leishmania* spp., retinol acetate was used as the reference molecule, leading to the discovery of various retinoids commonly used in dermatology. Isotretinoin, identified as a potential treatment for acne, was tested in vitro and demonstrated a strong trypanocidal effect at nanomolar concentrations [307]. In their study, Reigada et al. [308] highlighted the use of the anthracene–putrescine conjugate (Ant4) as a reference compound in LiSiCA v1.0, which blocks polyamine uptake in cancer cells. Furthermore, they found that antipsychotic drugs such as promazine, chlorpromazine, and clomipramine acted as inhibitors of putrescine uptake, showing strong trypanocidal activity.

## 8. Conclusion and Future Prospects

*Trypanosoma* and *Leishmania* species are kinetoplastids with complex life cycles, diverse transmission pathways, and harmful effects on humans and animals. Treatments for leishmaniasis, African sleeping sickness, and Chagas disease have historically progressed from empirical approaches to more advanced therapies. However, the development of drug resistance continues to be a significant challenge, requiring continued investigation into underlying mechanisms. Developing novel therapeutic approaches has been aided by understanding the molecular mechanisms underlying drug resistance in trypanosomatids. Novel drug targets have been identified due to advancements in genomic, transcriptomic, and proteomic technologies.

AI is set to significantly reshape how the pathogenesis of trypanosomatids is understood by enabling deeper integration of multiomics data at an unprecedented scale. Machine learning models can uncover subtle host–parasite interactions, identify virulence factors, and predict disease progression patterns that are not easily discernible through conventional analysis. AI‐driven structural biology tools will accelerate the characterization of parasite proteins, revealing novel mechanisms of immune evasion, antigenic variation, and intracellular survival. In addition, predictive modeling of host immune responses may help clarify why disease manifestations vary across populations, thereby refining our understanding of parasite adaptability and pathogenicity.

However, the effectiveness of AI tools will depend on data quality, infrastructure, and ethical governance. Moving forward, it would thus be helpful to consider interdisciplinary strategies that combine traditional approaches with AI technologies. With this, researchers and clinicians can improve treatment protocols, find new drugs, and create cutting‐edge diagnostic tools. Reducing the global burden of infections and improving health outcomes for impacted populations require sustained interdisciplinary collaboration and investment in emerging technologies. Emerging concepts of multidisciplinarity emphasize a shift toward systems‐level understanding of trypanosomatid biology, integrating metabolism, population dynamics, genetic exchange, and environmental drivers. Incorporating these perspectives into research and control strategies will be critical for developing sustainable interventions against these complex and evolving pathogens.

## Author Contributions

A.K.D.: study conception, writing–original draft, and review and editing; C.M.A., J.A.A.Y., A.F.A., B.K.G., A.A., Z.L.A., G.D., S.M.A‐T., N.K‐A.Q., J.H.N.O., S.K.L., and W.E.: writing–original draft and review and editing.

## Funding

This study was not supported by grant from any institution or government.

## Conflicts of Interest

The authors declare no conflicts of interest.

## Data Availability

All data generated and used in the study can be found in this manuscript.

## References

[1] Stuart K. , Brun R. , Croft S. et al., Kinetoplastids: Related Protozoan Pathogens, Different Diseases, The Journal of Clinical Investigation. (2008) 118, no. 4, 1301–1310, 10.1172/jci33945. 18382742 PMC2276762

[2] Cavalier-Smith T. , Higher Classification and Phylogeny of Euglenozoa, European Journal of Protistology. (2016) 56, 250–276, 10.1016/j.ejop.2016.09.003. 27889663

[3] Solter L. F. , Becnel J. J. , and Vávra J. , Research Methods for Entomopathogenic Microsporidia and Other Protists, Manual of Techniques in Invertebrate Pathology. (2012) 12, 329–371.

[4] Simpson A. G. B. , Stevens J. R. , and Lukeš J. , Are Trypanosomes Monophyletic? A Controversy Resolved (Again), Trends in Parasitology. (2006) 4, no. 22, 168–174. 10.1016/j.pt.2006.02.006 16504583

[5] Rao S. P. S. , Barrett M. P. , Dranoff G. et al., Drug Discovery for Kinetoplastid Diseases: Future Directions, ACS Infectious Diseases. (2018) 5, no. 2, 152–157, 10.1021/acsinfecdis.8b00298. 30543391

[6] World Health Organization , Chagas Disease, 2024, https://www.who.int/news-room/facts-in-pictures/detail/chagas-disease.

[7] Nunes M. C. P. , Bern C. , Clark E. H. , Teixeira A. L. , and Molina I. , Clinical Features of Chagas Disease Progression and Severity, The Lancet Regional Health. Americas. (2024) 37, 10.1016/j.lana.2024.100832.

[8] Winters R. , Nguyen T. , and Waseem M. , Chagas Disease, StatPearls [Internet], 2025, StatPearls Publishing, Treasure Island, FL, https://www.ncbi.nlm.nih.gov/books/NBK459272/. 29083573

[9] Checchi F. , Funk S. , Chandramohan D. , Chappuis F. , and Haydon D. T. , The Impact of Passive Case Detection on the Transmission Dynamics of Gambiense Human African Trypanosomiasis, PLoS Neglected Tropical Diseases. (2018) 12, no. 4, 10.1371/journal.pntd.0006276. PMC5906023 29624584

[10] World Health Organization , Leishmaniasis, 2023, https://www.who.int/news-room/fact-sheets/detail/leishmaniasis.

[11] Anderson N. E. , Mubanga J. , Fevre E. M. et al., Characterisation of the Wildlife Reservoir Community for Human and Animal Trypanosomiasis in the Luangwa Valley, Zambia, PLoS Neglected Tropical Diseases. (2011) 5, no. 6, 10.1371/journal.pntd.0001211. PMC3119639 21713019

[12] Ofori J. A. , Bakari S. M. , Bah S. , Kolugu M. K. , Aning G. K. , and Awandare G. A. , A Longitudinal Two-Year Survey of the Prevalence of Trypanosomes in Domestic Cattle in Ghana by Massively Parallel Sequencing of Barcoded Amplicons, PLoS Neglected Tropical Diseases. (2022) 16, no. 4, 10.1371/journal.pntd.0010300. PMC9060370 35442960

[13] World Health Organization , Trypanosomiasis, Human African (Sleeping Sickness), 2025, https://www.who.int/news-room/fact-sheets/detail/trypanosomiasis-human-african-(sleeping-sickness.

[14] World Health Organization , Trypanosomiasis, Human African (Sleeping Sickness), 2023, https://www.who.int/news-room/fact-sheets/detail/trypanosomiasis-human-african-(sleeping-sickness.

[15] De Koning H. , The Drugs of Sleeping Sickness: Their Mechanisms of Action and Resistance, and A Brief History, Tropical Medicine and Infectious Disease. (2020) 5, no. 1, 10.3390/tropicalmed5010014. PMC7157662 31963784

[16] Ebiloma G. U. , Alhejeli A. , and de Koning H. P. , Interventions for Neglected Diseases Caused by Kinetoplastid Parasites: A One Health Approach to Drug Discovery, Development, and Deployment, Pharmaceuticals. (2025) 18, no. 9, 10.3390/ph18091415. PMC12473039 41011282

[17] Chanda K. , An Overview on the Therapeutics of Neglected Infectious Diseases—Leishmaniasis and Chagas Diseases, Frontiers in Chemistry. (2021) 9. 10.3389/fchem.2021.622286 PMC7994601 33777895

[18] Horn D. , Antigenic Variation in African Trypanosomes, Molecular and Biochemical Parasitology. (2014) 195, no. 2, 123–129, 10.1016/j.molbiopara.2014.05.001. 24859277 PMC4155160

[19] Garg N. J. , An Update on Vaccines Against Trypanosoma cruzi and Chagas Disease, Pathogens. (2025) 14, no. 2, 10.3390/pathogens14020124. PMC11857938 40005501

[20] Li W. , Zhang H. , Assaraf Y. G. et al., Overcoming ABC Transporter-Mediated Multidrug Resistance: Molecular Mechanisms and Novel Therapeutic Drug Strategies, Drug Resistance Updates. (2016) 27, 14–29, 10.1016/j.drup.2016.05.001. 27449595

[21] Allemailem K. S. , Recent Advances in Understanding the Molecular Mechanisms of Multidrug Resistance and Novel Approaches of CRISPR/Cas9-Based Genome-Editing to Combat This Health Emergency, International Journal of Nanomedicine. (2024) 19, 1125–1143, 10.2147/ijn.s453566. 38344439 PMC10859101

[22] Visan A. I. and Negut I. , Integrating Artificial Intelligence for Drug Discovery in the Context of Revolutionizing Drug Delivery, Life. (2024) 14, no. 2, 10.3390/life14020233. PMC10890405 38398742

[23] Malheiro V. , Santos B. , Figueiras A. , and Mascarenhas-Melo F. , The Potential of Artificial Intelligence in Pharmaceutical Innovation: From Drug Discovery to Clinical Trials, Pharmaceuticals. (2025) 18, no. 6, 10.3390/ph18060788. PMC12195710 40573185

[24] Fallatah D. I. and Adekola H. A. , Digital Epidemiology: Harnessing Big Data for Early Detection and Monitoring of Viral Outbreaks, Infection Prevention in Practice. (2024) 6, no. 3, 10.1016/j.infpip.2024.100382. PMC11292357 39091623

[25] Qasrawi R. , Issa G. , Thwib S. et al., The Role of Machine Learning in Infectious Disease Early Detection and Prediction in the MENA Region: A Systematic Review, Informatics in Medicine Unlocked. (2025) 56, 10.1016/j.imu.2025.101651.

[26] Sundar S. , The Story of Elimination of Visceral Leishmaniasis (Kala-Azar) in India-Challenges towards Sustainment, PLoS Neglected Tropical Diseases. (August 2025) 19, no. 8, 10.1371/journal.pntd.0013321. PMC12364345 40828749

[27] Berriman M. , Ghedin E. , Hertz-Fowler C. et al., The Genome of the African Trypanosome Trypanosoma brucei, Science. (2005) 309, no. 5733, 416–422, 10.1126/science.1112642. 16020726

[28] El-Sayed N. M. , Myler P. J. , Bartholomeu D. C. et al., The Genome Sequence of Trypanosoma cruzi, Etiologic Agent of Chagas Disease, Science. (2005) 309, no. 5733, 409–415, 10.1126/science.1112631. 16020725

[29] Ivens A. C. , Peacock C. S. , Worthey E. A. et al., The Genome of the Kinetoplastid Parasite, Leishmania Major, Science. (2005) 309, no. 5733, 436–442, 10.1126/science.1112680. 16020728 PMC1470643

[30] Morand S. , Renggli C. K. , Roditi I. , and Vassella E. , MAP Kinase Kinase 1 (MKK1) is Essential for Transmission of Trypanosoma brucei by Glossina Morsitans, Molecular and Biochemical Parasitology. (2012) 186, no. 1, 73–76, 10.1016/j.molbiopara.2012.09.001. 22985893

[31] Borst P. , Antigenic Variation and Allelic Exclusion, Cell. (2002) 109, no. 1, 5–8, 10.1016/s0092-8674(02)00711-0. 11955440

[32] Pays E. , Vanhamme L. , and Pérez-Morga D. , Antigenic Variation in Trypanosoma brucei: Facts, Challenges and Mysteries, Current Opinion in Microbiology. (2004) 7, no. 4, 369–374, 10.1016/j.mib.2004.05.001. 15288623

[33] dos Santos S. L. , Freitas L. M. , Lobo F. P. et al., The MASP Family of Trypanosoma cruzi: Changes in Gene Expression and Antigenic Profile During the Acute Phase of Experimental Infection, 2012. 10.1371/journal.pntd.0001779 PMC3419193 22905275

[34] Rubin S. S. C. and Schenkman S. , T Rypanosoma cruzi Trans‐Sialidase as a Multifunctional Enzyme in C Hagas’ Disease, Cellular Microbiology. (2012) 14, no. 10, 1522–1530, 10.1111/j.1462-5822.2012.01831.x. 22747789

[35] Li Z.-H. , Alvarez V. E. , De Gaudenzi J. G. et al., Hyperosmotic Stress Induces Aquaporin-Dependent Cell Shrinkage, Polyphosphate Synthesis, Amino Acid Accumulation, and Global Gene Expression Changes in Trypanosoma cruzi, Journal of Biological Chemistry. (2011) 286, no. 51, 43959–43971, 10.1074/jbc.m111.311530. 22039054 PMC3243512

[36] Haynes C. L. F. , Ameloot P. , Remaut H. et al., Production, Purification and Crystallization of a {\it trans}-Sialidase From {\it Trypanosoma vivax}, Acta Crystallographica Section F. (2015) 71, no. 5, 577–585, 10.1107/S2053230X15002496. PMC4427168 25945712

[37] Yao C. , Gaur Dixit U. , Barker J. H. et al., Attenuation of Leishmania Infantum Chagasi Metacyclic Promastigotes by Sterol Depletion, Infection and Immunity. (2013) 81, no. 7, 2507–2517, 10.1128/iai.00214-13. 23630964 PMC3697599

[38] Schnaufer A. , Sbicego S. , and Blum B. , Antimycin A Resistance in a Mutant Leishmania Tarentolae Strain is Correlated to a Point Mutation in the Mitochondrial Apocytochrome b Gene, Current Genetics. (2000) 37, no. 4, 234–241, 10.1007/s002940050525. 10803885

[39] Surma M. A. , Szczepaniak A. , and Króliczewski J. , Comparative Studies on Detergent-Assisted Apocytochrome b6 Reconstitution Into Liposomal Bilayers Monitored by Zetasizer Instruments, PLoS One. (2014) 9, no. 11, 10.1371/journal.pone.0111341. PMC4244035 25423011

[40] Di Rago J. P. and Colson A. M. , Molecular Basis for Resistance to Antimycin and Diuron, Q-Cycle Inhibitors Acting at the Qi Site in the Mitochondrial Ubiquinol-Cytochrome c Reductase in *Saccharomyces cerevisiae* , Journal of Biological Chemistry. (1988) 263, no. 25, 12564–12570, 10.1016/s0021-9258(18)37792-5. 2842335

[41] Briggs E. , Hamilton G. , Crouch K. , Lapsley C. , and McCulloch R. , Genome-Wide Mapping Reveals Conserved and Diverged R-Loop Activities in the Unusual Genetic Landscape of the African Trypanosome Genome, Nucleic Acids Research. (2018) 46, no. 22, 11789–11805, 10.1093/nar/gky928. 30304482 PMC6294496

[42] Hutchinson S. , Foulon S. , Crouzols A. et al., The Establishment of Variant Surface Glycoprotein Monoallelic Expression Revealed by Single-Cell RNA-Seq of Trypanosoma brucei in the Tsetse Fly Salivary Glands, PLoS Pathogens. (2021) 17, no. 9, 10.1371/journal.ppat.1009904. PMC8509897 34543350

[43] Figueiredo L. M. , Cross G. A. , and Janzen C. J. , Epigenetic Regulation in African Trypanosomes: A New Kid on the Block, Nature Reviews Microbiology. (2009) 7, no. 7, 504–513, 10.1038/nrmicro2149. 19528957

[44] Szempruch A. J. , Dennison L. , Kieft R. , Harrington J. M. , and Hajduk S. L. , Sending a Message: Extracellular Vesicles of Pathogenic Protozoan Parasites, Nature Reviews Microbiology. (2016) 14, no. 11, 669–675, 10.1038/nrmicro.2016.110. 27615028

[45] Atayde V. D. , da Silva Lira Filho A. , Chaparro V. et al., Exploitation of the Leishmania Exosomal Pathway by Leishmania RNA Virus 1, Nature Microbiology. (2019) 4, no. 4, 714–723, 10.1038/s41564-018-0352-y. 30692670

[46] Beneke T. , Madden R. , Makin L. , Valli J. , Sunter J. , and Gluenz E. , A CRISPR Cas9 High-Throughput Genome Editing Toolkit for Kinetoplastids, Royal Society Open Science. (2017) 4, no. 5, 10.1098/rsos.170095. PMC5451818 28573017

[47] Damianou A. , Burge R. J. , Catta-Preta C. M. et al., Essential Roles for Deubiquitination in Leishmania Life Cycle Progression, PLoS Pathogens. (2020) 16, no. 6, 10.1371/journal.ppat.1008455. PMC7319358 32544189

[48] Steverding D. , The History of African Trypanosomiasis, Parasites & Vectors. (2008) 1, 1–8, 10.1186/1756-3305-1-3. 18275594 PMC2270819

[49] Davis C. N. , Rock K. S. , and Keeling M. J. , Human African Trypanosomiasis: Current Status and Eradication Efforts, 2020, CABI Reviews, 10.1079/PAVSNNR202015028.

[50] Holanda-Freitas I. T. , do Carmo Cupertino M. , dos Santos E. C. , Oliveira L. , Geller M. , and Siqueira-Batista R. , Human African Trypanosomiasis: Current Standing and Challenges, Revista de Patologia Tropical/Journal of Tropical Pathology. (2020) 49, no. 3.

[51] Hasker E. , Hope A. , and Bottieau E. , Gambiense Human African Trypanosomiasis: The Bumpy Road to Elimination, Current Opinion in Infectious Diseases. (2022) 35, no. 5, 384–389, https://journals.lww.com/co-infectiousdiseases/fulltext/2022/10000/gambiense_human_african_trypanosomiasis__the_bumpy.3.aspx, 10.1097/qco.0000000000000860. 35942856 PMC9553258

[52] Álvarez-Rodríguez A. , Jin B.-K. , Radwanska M. , and Magez S. , Recent Progress in Diagnosis and Treatment of Human African Trypanosomiasis Has Made the Elimination of This Disease a Realistic Target by 2030, Frontiers of Medicine. (2022) 9, 10.3389/fmed.2022.1037094. PMC9669443 36405602

[53] Eperon G. , Balasegaram M. , Potet J. , Mowbray C. , Valverde O. , and Chappuis F. , Treatment Options for Second-Stage Gambiense Human African Trypanosomiasis, Expert Review of Anti-Infective Therapy. (2014) 12, no. 11, 1407–1417, 10.1586/14787210.2014.959496. 25204360 PMC4743611

[54] Steverding D. , The Development of Drugs for Treatment of Sleeping Sickness: A Historical Review, Parasites & Vectors. (2010) 3, 1–9, 10.1186/1756-3305-3-15. 20219092 PMC2848007

[55] Brun R. , Schumacher R. , Schmid C. , Kunz C. , and Burri C. , The Phenomenon of Treatment Failures in Human African Trypanosomiasis, Tropical Medicine and International Health. (2001) 6, no. 11, 906–914, 10.1046/j.1365-3156.2001.00775.x. 11703845

[56] Okada T. and InuI T. , Development of Therapeutic Agents for Human African Trypanosomiasis, Translational and Regulatory Sciences. (2021) 3, no. 2, 43–50, 10.33611/trs.2021-006.

[57] Fairlamb A. H. and Horn D. , Melarsoprol Resistance in African Trypanosomiasis, Trends in Parasitology. (2018) 34, no. 6, 481–492, 10.1016/j.pt.2018.04.002. 29705579

[58] Güran M. , An Overview of Leishmaniasis: Historic to Future Perspectives, Vectors and Vector-Borne Zoonotic Diseases. (2018) .

[59] Steverding D. , The History of Leishmaniasis, Parasites & Vectors. (2017) 10, 1–10, 10.1186/s13071-017-2028-5. 28202044 PMC5312593

[60] Thomaz-Soccol V. , Lanotte G. , Rioux J. A. , Pratlong F. , Martini-Dumas A. , and Serres E. , Monophyletic Origin of the Genus Leishmania Ross, 1903, Annales de Parasitologie Humaine et Comparee. (1993) 68, no. 2, 107–108, 10.1051/parasite/1993682107. 8215109

[61] Momen H. and Cupolillo E. , Speculations on the Origin and Evolution of the Genus Leishmania, Memórias do Instituto Oswaldo Cruz. (2000) 95, no. 4, 583–588, 10.1590/s0074-02762000000400023. 10904419

[62] Knight C. A. , Harris D. R. , Alshammari S. O. , Gugssa A. , Young T. , and Lee C. M. , Leishmaniasis: Recent Epidemiological Studies in the Middle East, Frontiers in Microbiology. (2023) 13, 10.3389/fmicb.2022.1052478. PMC9932337 36817103

[63] Dorlo T. P. C. , Balasegaram M. , Beijnen J. H. , and de Vries P. J. , Miltefosine: A Review of its Pharmacology and Therapeutic Efficacy in the Treatment of Leishmaniasis, Journal of Antimicrobial Chemotherapy. (2012) 67, no. 11, 2576–2597, 10.1093/jac/dks275. 22833634

[64] Olias-Molero A. I. , de la Fuente C. , Cuquerella M. , Torrado J. J. , and Alunda J. M. , Antileishmanial Drug Discovery and Development: Time to Reset the Model?, Microorganisms. (2021) 9, no. 12, 10.3390/microorganisms9122500. PMC8703564 34946102

[65] Croft S. L. and Yardley V. , Chemotherapy of Leishmaniasis, Current Pharmaceutical Design. (2002) 8, no. 4, 319–342, 10.2174/1381612023396258. 11860369

[66] Barratt M. J. and Frail D. E. , Drug Repositioning: Bringing New Life to Shelved Assets and Existing Drugs, 2012, John Wiley & Sons.

[67] Pradhan S. , Schwartz R. A. , Patil A. , Grabbe S. , and Goldust M. , Treatment Options for Leishmaniasis, Clinical and Experimental Dermatology. (2022) 47, no. 3, 516–521, 10.1111/ced.14919. 34480806

[68] Majumder N. , Banerjee A. , and Saha S. , A Review on New Natural and Synthetic Anti-Leishmanial Chemotherapeutic Agents and Current Perspective of Treatment Approaches, Acta Tropica. (2023) 240, 10.1016/j.actatropica.2023.106846. 36720335

[69] Davidson R. N. , den Boer M. , and Ritmeijer K. , Paromomycin, Transactions of the Royal Society of Tropical Medicine and Hygiene. (2009) 103, no. 7, 653–660, 10.1016/j.trstmh.2008.09.008. 18947845

[70] Graf F. E. , Ludin P. , Wenzler T. et al., Aquaporin 2 Mutations in Trypanosoma brucei Gambiense Field Isolates Correlate With Decreased Susceptibility to Pentamidine and Melarsoprol, PLoS Neglected Tropical Diseases. (2013) 7, no. 10, 10.1371/journal.pntd.0002475. PMC3794916 24130910

[71] Mesu V. K. B. K. , Kalonji W. M. , Bardonneau C. et al., Oral Fexinidazole for Late-Stage African Trypanosoma brucei Gambiense Trypanosomiasis: a Pivotal Multicentre, Randomised, Non-Inferiority Trial, Lancet (London, England). (2018) 391, no. 10116, 144–154, 10.1016/S0140-6736(17)32758-7. 29113731

[72] Wyllie S. , Foth B. J. , Kelner A. , Sokolova A. Y. , Berriman M. , and Fairlamb A. H. , Nitroheterocyclic Drug Resistance Mechanisms in Trypanosoma brucei, Journal of Antimicrobial Chemotherapy. (2016) 71, no. 3, 625–634, 10.1093/JAC/DKV376. 26581221 PMC4743696

[73] Ponte-Sucre A. , Gamarro F. , Dujardin J.-C. et al., Drug Resistance and Treatment Failure in Leishmaniasis: A 21st Century Challenge, PLoS Neglected Tropical Diseases. (2017) 11, no. 12, 10.1371/journal.pntd.0006052. PMC5730103 29240765

[74] Burza S. , Croft S. L. , and Boelaert M. , Leishmaniasis, Lancet (London, England). (2018) 392, no. 10151, 951–970, 10.1016/S0140-6736(18)31204-2. 30126638

[75] Mondelaers A. , Sanchez-Cañete M. P. , Hendrickx S. et al., Genomic and Molecular Characterization of Miltefosine Resistance in Leishmania Infantum Strains With Either Natural or Acquired Resistance Through Experimental Selection of Intracellular Amastigotes, PLoS One. (2016) 11, no. 4, 10.1371/journal.pone.0154101. PMC4849676 27123924

[76] Goodwin L. G. , Pentostam®(Sodium Stibogluconate); A 50-Year Personal Reminiscence, Transactions of the Royal Society of Tropical Medicine and Hygiene. (1995) 89, no. 3, 339–341, 10.1016/0035-9203(95)90572-3. 7660456

[77] Frézard F. , Demicheli C. , and Ribeiro R. R. , Pentavalent Antimonials: New Perspectives for Old Drugs, Molecules. (2009) 14, no. 7, 2317–2336, 10.3390/molecules14072317. 19633606 PMC6254722

[78] Burki T. , East African Countries Struggle With Visceral Leishmaniasis, The Lancet. (2009) 374, no. 9687, 371–372, 10.1016/s0140-6736(09)61401-x. 19655434

[79] Sundar S. , More D. K. , Singh M. K. et al., Failure of Pentavalent Antimony in Visceral Leishmaniasis in India: Report From the Center of the Indian Epidemic, Clinical Infectious Diseases. (2000) 31, no. 4, 1104–1107, 10.1086/318121. 11049798

[80] Mishra J. , Saxena A. , and Singh S. , Chemotherapy of Leishmaniasis: Past, Present and Future, Current Medicinal Chemistry. (2007) 14, no. 10, 1153–1169, 10.2174/092986707780362862. 17456028

[81] Frézard F. , Martins P. S. , Barbosa M. C. M. et al., New Insights Into the Chemical Structure and Composition of the Pentavalent Antimonial Drugs, Meglumine Antimonate and Sodium Stibogluconate, Journal of Inorganic Biochemistry. (2008) 102, no. 4, 656–665, 10.1016/j.jinorgbio.2007.10.010. 18061680

[82] Matos A. P. S. , Viçosa A. L. , Ré M.-I. , Ricci-Júnior E. , and Holandino C. , A Review of Current Treatments Strategies Based on Paromomycin for Leishmaniasis, Journal of Drug Delivery Science and Technology. (2020) 57, 10.1016/j.jddst.2020.101664.

[83] Goodwin L. G. , The Chemotherapy of Experimental Leishmaniasis. II. A Dose Response Curve for the Activity of Sodium Stibogluconate, 1945. 10.1016/0035-9203(45)90004-6 20293985

[84] Sereno D. , Cavaleyra M. , Zemzoumi K. , Maquaire S. , Ouaissi A. , and Lemesre J. L. , Axenically Grown Amastigotes of Leishmania Infantum Used as an In Vitro Model to Investigate the Pentavalent Antimony Mode of Action, Antimicrobial Agents and Chemotherapy. (1998) 42, no. 12, 3097–3102, 10.1128/aac.42.12.3097. 9835497 PMC106005

[85] Shaked-Mishan P. , Ulrich N. , Ephros M. , and Zilberstein D. , Novel Intracellular SbV Reducing Activity Correlates With Antimony Susceptibility in Leishmania donovani, Journal of Biological Chemistry. (2001) 276, no. 6, 3971–3976, 10.1074/jbc.m005423200. 11110784

[86] Yan S. , Li F. , Ding K. , and Sun H. , Reduction of Pentavalent Antimony by Trypanothione and Formation of a Binary and Ternary Complex of Antimony (III) and Trypanothione, JBIC, Journal of Biological Inorganic Chemistry. (2003) 8, no. 6, 689–697, 10.1007/s00775-003-0468-1. 12827457

[87] Légaré D. , Richard D. , Mukhopadhyay R. et al., The Leishmania ATP-Binding Cassette Protein PGPA Is an Intracellular Metal-Thiol Transporter ATPase, Journal of Biological Chemistry. (2001) 276, no. 28, 26301–26307, 10.1074/jbc.m102351200. 11306588

[88] Haldar A. K. , Sen P. , and Roy S. , Use of Antimony in the Treatment of Leishmaniasis: Current Status and Future Directions, Molecular Biology International. (2011) 2011, no. 1, 571242–23, 10.4061/2011/571242. 22091408 PMC3196053

[89] Mandal G. , Wyllie S. , Singh N. , Sundar S. , Fairlamb A. H. , and Chatterjee M. , Increased Levels of Thiols Protect Antimony Unresponsive Leishmania donovani Field Isolates Against Reactive Oxygen Species Generated by Trivalent Antimony, Parasitology. (2007) 134, no. 12, 1679–1687, 10.1017/s0031182007003150. 17612420 PMC3409873

[90] Chakraborty A. K. and Majumder H. K. , Mode of Action of Pentavalent Antimonials: Specific Inhibition of Type I DNA Topoisomerase of Leishmaniadonovani, Biochemical and Biophysical Research Communications. (1988) 152, no. 2, 605–611, 10.1016/s0006-291x(88)80081-0. 2835038

[91] Herman J. D. , Gallalee J. V. , and Best J. M. , Sodium Stibogluconate (Pentostam) Inhibition of Glucose Catabolism via the Glycolytic Pathway, and Fatty Acid β-Oxidation in Leishmania Mexicana Amastigotes, Biochemical Pharmacology. (1987) 36, no. 2, 197–201, 10.1016/0006-2952(87)90689-7. 3028425

[92] Sundar S. and Chatterjee M. , Visceral Leishmaniasis-Current Therapeutic Modalities, Indian Journal of Medical Research. (2006) 123, no. 3. 16778315

[93] Kuhlmann F. M. and Fleckenstein J. M. , Antiparasitic Agents, Infectious Diseases, 2-Volume Set, 2017, Elsevier, 1345–1372.

[94] Mehta A. and Shaha C. , Mechanism of Metalloid-Induced Death in Leishmania spp.: Role of Iron, Reactive Oxygen Species, Ca2+, and Glutathione, Free Radical Biology and Medicine. (2006) 40, no. 10, 1857–1868, 10.1016/j.freeradbiomed.2006.01.024. 16678023

[95] Castillo E. , A Dea-Ayuela M. , Bolás-Fernández F. , Rangel M. , and E Gonzalez-Rosende M. , The Kinetoplastid Chemotherapy Revisited: Current Drugs, Recent Advances and Future Perspectives, Current Medicinal Chemistry. (2010) 17, no. 33, 4027–4051. 20939823 10.2174/092986710793205345

[96] Reguera R. M. , Pérez-Pertejo Y. , Gutiérrez-Corbo C. et al., Current and Promising Novel Drug Candidates Against Visceral Leishmaniasis, Pure and Applied Chemistry. (2019) 91, no. 8, 1385–1404, 10.1515/pac-2018-1102.

[97] Mukhopadhyay R. , Dey S. , Xu N. et al., Trypanothione Overproduction and Resistance to Antimonials and Arsenicals in Leishmania, Proceedings of the National Academy of Sciences. (1996) 93, no. 19, 10383–10387, 10.1073/pnas.93.19.10383. PMC38393 8816809

[98] dos Santos Ferreira C. , Silveira Martins P. , Demicheli C. , Brochu C. , Ouellette M. , and Frézard F. , Thiol-Induced Reduction of Antimony (V) Into Antimony (III): A Comparative Study With Trypanothione, Cysteinyl-Glycine, Cysteine and Glutathione, Biometals. (2003) 16, no. 3, 441–446, 10.1023/a:1022823605068. 12680707

[99] Croft S. L. , Sundar S. , and Fairlamb A. H. , Drug Resistance in Leishmaniasis, Clinical Microbiology Reviews. (2006) 19, no. 1, 111–126, 10.1128/cmr.19.1.111-126.2006. 16418526 PMC1360270

[100] Marquis N. , Gourbal B. , Rosen B. P. , Mukhopadhyay R. , and Ouellette M. , Modulation in Aquaglyceroporin AQP1 Gene Transcript Levels in Drug‐Resistant Leishmania, Molecular Microbiology. (2005) 57, no. 6, 1690–1699, 10.1111/j.1365-2958.2005.04782.x. 16135234

[101] Ashutosh , Sundar S. , and Goyal N. , Molecular Mechanisms of Antimony Resistance in Leishmania, Journal of Medical Microbiology. (2007) 56, no. 2, 143–153, 10.1099/jmm.0.46841-0. 17244793

[102] Mandal S. , Maharjan M. , Singh S. , Chatterjee M. , and Madhubala R. , Assessing Aquaglyceroporin Gene Status and Expression Profile in Antimony-Susceptible And-Resistant Clinical Isolates of Leishmania donovani From India, Journal of Antimicrobial Chemotherapy. (2010) 65, no. 3, 496–507, 10.1093/jac/dkp468. 20067981

[103] Mukherjee A. , Padmanabhan P. K. , Singh S. et al., Role of ABC Transporter MRPA, γ-glutamylcysteine Synthetase and Ornithine Decarboxylase in Natural Antimony-Resistant Isolates of Leishmania donovani, Journal of Antimicrobial Chemotherapy. (2007) 59, no. 2, 204–211. 17213267 10.1093/jac/dkl494

[104] do Monte-Neto R. L. , Coelho A. C. , Raymond F. et al., Gene Expression Profiling and Molecular Characterization of Antimony Resistance in Leishmania Amazonensis, PLoS Neglected Tropical Diseases. (2011) 5, no. 5, 10.1371/journal.pntd.0001167. PMC3101167 21629719

[105] Friedheim E. A. H. , Mel B in the Treatment of Human Trypanosomiasis, The American Journal of Tropical Medicine. (1949) 1-29, no. 2, 173–180, 10.4269/ajtmh.1949.s1-29.173. 18116843

[106] Wilkinson S. R. and Kelly J. M. , Trypanocidal Drugs: Mechanisms, Resistance and New Targets, Expert Reviews in Molecular Medicine. (2009) 11, 10.1017/s1462399409001252. 19863838

[107] Carter N. S. and Fairlamb A. H. , Arsenical-Resistant Trypanosomes Lack an Unusual Adenosine Transporter, Nature. (1993) 361, no. 6408, 173–176, 10.1038/361173a0. 8421523

[108] Mäser P. , Sütterlin C. , Kralli A. , and Kaminsky R. , A Nucleoside Transporter From Trypanosoma brucei Involved in Drug Resistance, Science (New York, N.Y.). (1999) 285, no. 5425, 242–244, 10.1126/SCIENCE.285.5425.242. 10398598

[109] Baker N. , Glover L. , Munday J. C. et al., Aquaglyceroporin 2 Controls Susceptibility to Melarsoprol and Pentamidine in African Trypanosomes, Proceedings of the National Academy of Sciences. (2012) 109, no. 27, 10996–11001, 10.1073/pnas.1202885109. PMC3390834 22711816

[110] Delespaux V. and de Koning H. P. , Drugs and Drug Resistance in African Trypanosomiasis, Drug Resistance Updates. (2007) 10, no. 1–2, 30–50, 10.1016/j.drup.2007.02.004. 17409013

[111] Fairlamb A. H. , Henderson G. B. , and Cerami A. , Trypanothione Is the Primary Target for Arsenical Drugs Against African Trypanosomes, Proceedings of the National Academy of Sciences. (1989) 86, no. 8, 2607–2611, 10.1073/pnas.86.8.2607. PMC286966 2704738

[112] Fairlamb A. H. and Cerami A. , Metabolism and Functions of Trypanothione in the Kinetoplastida, Annual Review of Microbiology. (1992) 46, no. 1, 695–729, 10.1146/annurev.micro.46.1.695. 1444271

[113] Wang C. C. , Molecular Mechanisms and Therapeutic Approaches to the Treatment of African Trypanosomiasis, Annual Review of Pharmacology and Toxicology. (1995) 35, no. 1, 93–127, 10.1146/annurev.pharmtox.35.1.93. 7598514

[114] Matovu E. , Stewart M. L. , Geiser F. et al., Mechanisms of Arsenical and Diamidine Uptake and Resistance in Trypanosoma brucei, Eukaryotic Cell. (2003) 2, no. 5, 1003–1008, 10.1128/ec.2.5.1003-1008.2003. 14555482 PMC219364

[115] Bridges D. J. , Gould M. K. , Nerima B. , Mäser P. , Burchmore R. J. S. , and De Koning H. P. , Loss of the High-Affinity Pentamidine Transporter is Responsible for High Levels of Cross-Resistance Between Arsenical and Diamidine Drugs in African Trypanosomes, Molecular Pharmacology. (2007) 71, no. 4, 1098–1108, 10.1124/mol.106.031351. 17234896

[116] Shahi S. K. , Krauth‐Siegel R. L. , and Clayton C. E. , Overexpression of the Putative Thiol Conjugate Transporter TbMRPA Causes Melarsoprol Resistance in Trypanosoma brucei, Molecular Microbiology. (2002) 43, no. 5, 1129–1138, 10.1046/j.1365-2958.2002.02831.x. 11918801

[117] Molyneux D. H. , Control of Human Parasitic Diseases: Context and Overview, Advances in Parasitology. (2006) 61, 1–45. 16735161 10.1016/S0065-308X(05)61001-9

[118] Alvar J. , Croft S. , and Olliaro P. , Chemotherapy in the Treatment and Control of Leishmaniasis, Advances in Parasitology. (2006) 61, 223–274. 16735166 10.1016/S0065-308X(05)61006-8

[119] Crofts T. S. , Gasparrini A. J. , and Dantas G. , Next-Generation Approaches to Understand and Combat the Antibiotic Resistome, Nature Reviews Microbiology. (2017) 15, no. 7, 422–434, 10.1038/nrmicro.2017.28. 28392565 PMC5681478

[120] Berman J. D. , Badaro R. , Thakur C. P. et al., Efficacy and Safety of Liposomal Amphotericin B (AmBisome) for Visceral Leishmaniasis in Endemic Developing Countries, Bulletin of the World Health Organization. (1998) 76, no. 1. PMC2305623 9615494

[121] Meyerhoff A. , US Food and Drug Administration Approval of AmBisome (Liposomal Amphotericin B) for Treatment of Visceral Leishmaniasis, Clinical Infectious Diseases. (1999) 28, no. 1, 42–48, 10.1086/515085. 10028069

[122] Brajtburg J. and Bolard J. , Carrier Effects on Biological Activity of Amphotericin B, Clinical Microbiology Reviews. (1996) 9, no. 4, 512–531, 10.1128/cmr.9.4.512. 8894350 PMC172908

[123] Frézard F. , Aguiar M. M. G. , Ferreira L. A. M. et al., Liposomal Amphotericin B for Treatment of Leishmaniasis: From the Identification of Critical Physicochemical Attributes to the Design of Effective Topical and Oral Formulations, Pharmaceutics. (2022) 15, no. 1, 10.3390/pharmaceutics15010099. PMC9864876 36678729

[124] Saha A. K. , Mukherjee T. , and Bhaduri A. , Mechanism of Action of Amphotericin B on Leishmania donovani Promastigotes, Molecular and Biochemical Parasitology. (1986) 19, no. 3, 195–200, 10.1016/0166-6851(86)90001-0. 3736592

[125] Lamp-Freund M. T. , Ferreira V. F. N. , and Schreier S. , Mechanism of Inactivation of the Polyene Antibiotic Amphotericin B Evidence for Radical Formation in the Process of Autooxidation, Journal of Antibiotics. (1985) 38, no. 6, 753–757, 10.7164/antibiotics.38.753. 2991182

[126] Mesa-Arango A. C. , Trevijano-Contador N. , Román E. et al., The Production of Reactive Oxygen Species is a Universal Action Mechanism of Amphotericin B against Pathogenic Yeasts and Contributes to the Fungicidal Effect of This Drug, Antimicrobial Agents and Chemotherapy. (2014) 58, no. 11, 6627–6638, 10.1128/aac.03570-14. 25155595 PMC4249417

[127] Mbongo N. , Loiseau P. M. , Billion M. A. , and Robert-Gero M. , Mechanism of Amphotericin B Resistance in Leishmania donovani Promastigotes, Antimicrobial Agents and Chemotherapy. (1998) 42, no. 2, 352–357, 10.1128/aac.42.2.352. 9527785 PMC105413

[128] McGonigle S. , Dalton J. P. , and James E. R. , Peroxidoxins: A New Antioxidant Family, Parasitology Today. (1998) 14, no. 4, 139–145, 10.1016/s0169-4758(97)01211-8. 17040731

[129] Purkait B. , Kumar A. , Nandi N. et al., Mechanism of Amphotericin B Resistance in Clinical Isolates of Leishmania donovani, Antimicrobial Agents and Chemotherapy. (2012) 56, no. 2, 1031–1041, 10.1128/aac.00030-11. 22123699 PMC3264217

[130] Kaur G. and Rajput B. , Comparative Analysis of the Omics Technologies Used to Study Antimonial, Amphotericin B, and Pentamidine Resistance in Leishmania, Journal of Parasitology Research. (2014) 2014, no. 1, 11, 726328, 10.1155/2014/726328. 24900912 PMC4036598

[131] Jhingran A. , Chawla B. , Saxena S. , Barrett M. P. , and Madhubala R. , Paromomycin: Uptake and Resistance in Leishmania donovani, Molecular and Biochemical Parasitology. (2009) 164, no. 2, 111–117, 10.1016/j.molbiopara.2008.12.007. 19146886 PMC3039421

[132] Fernández M. M. , Malchiodi E. L. , and Algranati I. D. , Differential Effects of Paromomycin on Ribosomes of Leishmania Mexicana and Mammalian Cells, Antimicrobial Agents and Chemotherapy. (2011) 55, no. 1, 86–93, 10.1128/aac.00506-10. 20956601 PMC3019668

[133] Shalev M. , Kondo J. , Kopelyanskiy D. , Jaffe C. L. , Adir N. , and Baasov T. , Identification of the Molecular Attributes Required for Aminoglycoside Activity Against Leishmania, Proceedings of the National Academy of Sciences. (2013) 110, no. 33, 13333–13338, 10.1073/pnas.1307365110. PMC3746865 23898171

[134] Maarouf M. , de Kouchkovsky Y. , Brown S. , Petit P. X. , and Robert-Gero M. , In Vivointerference of Paromomycin With Mitochondrial Activity of Leishmania, Experimental Cell Research. (1997) 232, no. 2, 339–348, 10.1006/excr.1997.3500. 9168810

[135] Fong D. , Chan M. M. , Rodriguez R. , Gately L. J. , Berman J. D. , and Grogl M. , Paromomycin Resistance in Leishmania Tropica: Lack of Correlation With Mutation in the Small Subunit Ribosomal RNA Gene, The American Journal of Tropical Medicine and Hygiene. (1994) 51, no. 6, 758–766, 10.4269/ajtmh.1994.51.758. 7810808

[136] Chawla B. , Jhingran A. , Panigrahi A. , Stuart K. D. , and Madhubala R. , Paromomycin Affects Translation and Vesicle-Mediated Trafficking as Revealed by Proteomics of Paromomycin–Susceptible–Resistant Leishmania donovani, PLoS One. (2011) 6, no. 10, 10.1371/journal.pone.0026660. PMC3203147 22046323

[137] Nagle A. S. , Khare S. , Kumar A. B. et al., Recent Developments in Drug Discovery for Leishmaniasis and Human African Trypanosomiasis, Chemical Reviews. (2014) 114, no. 22, 11305–11347, 10.1021/cr500365f. 25365529 PMC4633805

[138] Burri C. , Chappuis F. , and Brun R. , Human African Trypanosomiasis, 2014. 10.1016/S0140-6736(09)60829-1 19833383

[139] Baker N. , de Koning H. P. , Mäser P. , and Horn D. , Drug Resistance in African Trypanosomiasis: The Melarsoprol and Pentamidine Story, Trends in Parasitology. (2013) 29, no. 3, 110–118, 10.1016/j.pt.2012.12.005. 23375541 PMC3831158

[140] De Koning H. P. , Uptake of Pentamidine in Trypanosoma brucei Bruceiis Mediated by Three Distinct Transporters: Implications for Cross-Resistance With Arsenicals, Molecular Pharmacology. (2001) 59, no. 3, 586–592, 10.1016/s0026-895x(24)12250-x. 11179454

[141] Basselin M. , Denise H. , Coombs G. H. , and Barrett M. P. , Resistance to Pentamidine in Leishmania Mexicana Involves Exclusion of the Drug From the Mitochondrion, Antimicrobial Agents and Chemotherapy. (2002) 46, no. 12, 3731–3738, 10.1128/aac.46.12.3731-3738.2002. 12435669 PMC132791

[142] Bray P. G. , Barrett M. P. , Ward S. A. , and de Koning H. P. , Pentamidine Uptake and Resistance in Pathogenic Protozoa: Past, Present and Future, Trends in Parasitology. (2003) 19, no. 5, 232–239, 10.1016/s1471-4922(03)00069-2. 12763430

[143] de Koning H. P. , Anderson L. F. , Stewart M. , Burchmore R. J. S. , Wallace L. J. M. , and Barrett M. P. , The Trypanocide Diminazene Aceturate is Accumulated Predominantly Through the TbAT1 Purine Transporter: Additional Insights on Diamidine Resistance in African Trypanosomes, Antimicrobial Agents and Chemotherapy. (2004) 48, no. 5, 1515–1519, 10.1128/aac.48.5.1515-1519.2004. 15105099 PMC400564

[144] Basselin M. , Badet-Denisot M.-A. , and Robert-Gero M. , Modification of Kinetoplast DNA Minicircle Composition in Pentamidine-Resistant Leishmania, Acta Tropica. (1998) 70, no. 1, 43–61. 9707364 10.1016/s0001-706x(98)00007-2

[145] Riou G. and Benard J. , Berenil Induces the Complete Loss of Kinetoplast DNA Sequences in Trypanosoma Equiperdum, Biochemical and Biophysical Research Communications. (1980) 96, no. 1, 350–354, 10.1016/0006-291x(80)91221-8. 7437039

[146] Shapiro T. A. and Englund P. T. , Selective Cleavage of Kinetoplast DNA Minicircles Promoted by Antitrypanosomal Drugs, Proceedings of the National Academy of Sciences. (1990) 87, no. 3, 950–954, 10.1073/pnas.87.3.950. PMC53387 2153980

[147] Thomas J. A. , Baker N. , Hutchinson S. et al., Insights Into Antitrypanosomal Drug Mode-of-Action from Cytology-Based Profiling, PLoS Neglected Tropical Diseases. (2018) 12, no. 11, 10.1371/journal.pntd.0006980. PMC6283605 30475806

[148] Portugal J. , Berenil Acts as a Poison of Eukaryotic Topoisomerase II, FEBS Letters. (1994) 344, no. 2–3, 136–138, 10.1016/0014-5793(94)00363-7. 8187871

[149] Basselin M. , Lawrence F. , and Robert-Gero M. , Pentamidine Uptake in Leishmania donovani and Leishmania amazonensis Promastigotes and Axenic Amastigotes, Biochemical Journal. (1996) 315, no. Pt 2, 631–634, 10.1042/bj3150631. 8615840 PMC1217243

[150] Jean-Moreno V. , Rojas R. , Goyeneche D. , Coombs G. H. , and Walker J. , Leishmania donovani: Differential Activities of Classical Topoisomerase Inhibitors and Antileishmanials Against Parasite and Host Cells at the Level of DNA Topoisomerase I and in Cytotoxicity Assays, Experimental Parasitology. (2006) 112, no. 1, 21–30, 10.1016/j.exppara.2005.08.014. 16293247

[151] Bernhard S. C. , Nerima B. , Mäser P. , and Brun R. , Melarsoprol-and Pentamidine-Resistant Trypanosoma brucei Rhodesiense Populations and Their Cross-Resistance, International Journal for Parasitology. (2007) 37, no. 13, 1443–1448, 10.1016/j.ijpara.2007.05.007. 17602691

[152] Munday J. C. , Settimo L. , and De Koning H. P. , Transport Proteins Determine Drug Sensitivity and Resistance in a Protozoan Parasite, Trypanosoma brucei, Frontiers in Pharmacology. (2015) 6, 10.3389/fphar.2015.00032. PMC4356943 25814953

[153] Carruthers L. V. , Munday J. C. , Ebiloma G. U. et al., Diminazene Resistance in Trypanosoma Congolense is Not Caused by Reduced Transport Capacity But Associated With Reduced Mitochondrial Membrane Potential, Molecular Microbiology. (2021) 116, no. 2, 564–588, 10.1111/mmi.14733. 33932053

[154] Coelho A. C. , Beverley S. M. , and Cotrim P. C. , Functional Genetic Identification of PRP1, an ABC Transporter Superfamily Member Conferring Pentamidine Resistance in Leishmania Major, Molecular and Biochemical Parasitology. (2003) 130, no. 2, 83–90, 10.1016/s0166-6851(03)00162-2. 12946844

[155] Coelho A. C. , Messier N. , Ouellette M. , and Cotrim P. C. , Role of the ABC Transporter PRP1 (ABCC7) in Pentamidine Resistance in Leishmania amastigotes, Antimicrobial Agents and Chemotherapy. (2007) 51, no. 8, 3030–3032, 10.1128/aac.00404-07. 17452480 PMC1932501

[156] Alsford S. , Eckert S. , Baker N. et al., High-throughput Decoding of Antitrypanosomal Drug Efficacy and Resistance, Nature. (2012) 482, no. 7384, 232–236, 10.1038/nature10771. 22278056 PMC3303116

[157] Perez Anton E. , Dujeancourt-Henry A. , Rotureau B. , and Glover L. , A CRISPR-Based Diagnostic Tool to Survey Drug Resistance in Human African Trypanosomiasis, Antimicrobial Agents and Chemotherapy. (2025) 69, no. 12, e00933–25, 10.1128/aac.00933-25. 41251373 PMC12691586

[158] Soto J. and Soto P. , Miltefosine: Oral Treatment of Leishmaniasis, Expert Review of Anti-Infective Therapy. (2006) 4, no. 2, 177–185, 10.1586/14787210.4.2.177. 16597200

[159] Pérez-Victoria F. J. , Sánchez-Cañete M. P. , Castanys S. , and Gamarro F. , Phospholipid Translocation and Miltefosine Potency Require Both L. Donovani Miltefosine Transporter and the New Protein LdRos3 in Leishmania Parasites, Journal of Biological Chemistry. (2006) 281, no. 33, 23766–23775, 10.1074/jbc.m605214200. 16785229

[160] Rakotomanga M. , Saint-Pierre-Chazalet M. , and Loiseau P. M. , Alteration of Fatty Acid and Sterol Metabolism in Miltefosine-Resistant Leishmania donovani Promastigotes and Consequences for Drug-Membrane Interactions, Antimicrobial Agents and Chemotherapy. (2005) 49, no. 7, 2677–2686, 10.1128/aac.49.7.2677-2686.2005. 15980336 PMC1168669

[161] Rakotomanga M. , Blanc S. , Gaudin K. , Chaminade P. , and Loiseau P. M. , Miltefosine Affects Lipid Metabolism in Leishmania donovani Promastigotes, Antimicrobial Agents and Chemotherapy. (2007) 51, no. 4, 1425–1430, 10.1128/aac.01123-06. 17242145 PMC1855451

[162] Luque-Ortega J. R. and Rivas L. , Miltefosine (Hexadecylphosphocholine) Inhibits Cytochrome C Oxidase in Leishmania donovani Promastigotes, Antimicrobial Agents and Chemotherapy. (2007) 51, no. 4, 1327–1332, 10.1128/aac.01415-06. 17283192 PMC1855476

[163] Pinto-Martinez A. K. , Rodriguez-Durán J. , Serrano-Martin X. , Hernandez-Rodriguez V. , and Benaim G. , Mechanism of Action of Miltefosine on Leishmania donovani Involves the Impairment of Acidocalcisome Function and the Activation of the Sphingosine-Dependent Plasma Membrane Ca2+ Channel, Antimicrobial Agents and Chemotherapy. (2018) 62, no. 1, 10–1128, 10.1128/aac.01614-17. PMC5740361 29061745

[164] Verma N. K. and Dey C. S. , Possible Mechanism of Miltefosine-Mediated Death of Leishmania donovani, Antimicrobial Agents and Chemotherapy. (2004) 48, no. 8, 3010–3015, 10.1128/aac.48.8.3010-3015.2004. 15273114 PMC478494

[165] Wadhone P. , Maiti M. , Agarwal R. , Kamat V. , Martin S. , and Saha B. , Miltefosine Promotes IFN-γ-Dominated Anti-Leishmanial Immune Response, The Journal of Immunology. (2009) 182, no. 11, 7146–7154, 10.4049/jimmunol.0803859. 19454711

[166] Ghosh M. , Roy K. , and Roy S. , Immunomodulatory Effects of Antileishmanial Drugs, Journal of Antimicrobial Chemotherapy. (2013) 68, no. 12, 2834–2838, 10.1093/jac/dkt262. 23833177

[167] Pérez-Victoria F. J. , Gamarro F. , Ouellette M. , and Castanys S. , Functional Cloning of the Miltefosine Transporter: A Novel P-Type Phospholipid Translocase From Leishmania Involved in Drug Resistance, Journal of Biological Chemistry. (2003) 278, no. 50, 49965–49971. 14514670 10.1074/jbc.M308352200

[168] Pérez-Victoria J. M. , Pérez-Victoria F. J. , Parodi-Talice A. et al., Alkyl-Lysophospholipid Resistance in Multidrug-Resistant Leishmania Tropica and Chemosensitization by a Novel P-Glycoprotein-Like Transporter Modulator, Antimicrobial Agents and Chemotherapy. (2001) 45, no. 9, 2468–2474, 10.1128/aac.45.9.2468-2474.2001. 11502516 PMC90679

[169] Castanys‐Muñoz E. , Alder‐Baerens N. , Pomorski T. , Gamarro F. , and Castanys S. , A Novel ATP‐Binding Cassette Transporter From Leishmania is Involved in Transport of Phosphatidylcholine Analogues and Resistance to Alkyl‐Phospholipids, Molecular Microbiology. (2007) 64, no. 5, 1141–1153, 10.1111/j.1365-2958.2007.05653.x. 17542911

[170] Castanys-Munoz E. , Perez-Victoria J. M. , Gamarro F. , and Castanys S. , Characterization of an ABCG-Like Transporter From the Protozoan Parasite Leishmania With a Role in Drug Resistance and Transbilayer Lipid Movement, Antimicrobial Agents and Chemotherapy. (2008) 52, no. 10, 3573–3579, 10.1128/aac.00587-08. 18644961 PMC2565886

[171] Vergnes B. , Gourbal B. , Girard I. , Sundar S. , Drummelsmith J. , and Ouellette M. , A Proteomics Screen Implicates HSP83 and a Small Kinetoplastid Calpain-Related Protein in Drug Resistance in Leishmania donovani Clinical Field Isolates by Modulating Drug-Induced Programmed Cell Death, Molecular & Cellular Proteomics. (2007) 6, no. 1, 88–101, 10.1074/mcp.m600319-mcp200. 17050524

[172] Wegner D. H. and Rohwedder R. W. , The Effect of Nifurtimox in Acute Chagas’ Infection, Arzneimittel-Forschung. (1972) 22, no. 9, 1624–1635. 4630485

[173] Barrett M. P. , Burchmore R. J. S. , Stich A. et al., The Trypanosomiases, The Lancet. (2003) 362, no. 9394, 1469–1480. 10.1016/S0140-6736(03)14694-6 14602444

[174] Pepin J. and Milord F. , The Treatment of Human African Trypanosomiasis, Advances in Parasitology. (1994) 33, no. 10.1016, 60410–60418. 10.1016/s0065-308x(08)60410-8 8122565

[175] Pinazo M. J. , Forsyth C. , Losada I. et al., Efficacy and Safety of Fexinidazole for Treatment of Chronic Indeterminate Chagas Disease (FEXI-12): A Multicentre, Randomised, Double-Blind, Phase 2 Trial, The Lancet Infectious Diseases. (2024) 24, no. 4, 395–403, 10.1016/S1473-3099(23)00651-5. 38218194

[176] McCalla D. R. , Olive P. , Tu Y. , and Fan M. L. , Nitrofurazone-Reducing Enzymes in *E. coli* and Their Role in Drug Activation In Vivo, Canadian Journal of Microbiology. (1975) 21, no. 10, 1484–1491, 10.1139/m75-220. 136

[177] Wilkinson S. R. , Taylor M. C. , Horn D. , Kelly J. M. , and Cheeseman I. , A Mechanism for Cross-Resistance to Nifurtimox and Benznidazole in Trypanosomes, Proceedings of the National Academy of Sciences. (2008) 105, no. 13, 5022–5027, 10.1073/pnas.0711014105. PMC2278226 18367671

[178] Wyllie S. , Patterson S. , Stojanovski L. et al., The Anti-Trypanosome Drug Fexinidazole Shows Potential for Treating Visceral Leishmaniasis, Science Translational Medicine. (2012) 4, no. 119, 10.1126/scitranslmed.3003326. PMC3457684 22301556

[179] Sokolova A. Y. , Wyllie S. , Patterson S. , Oza S. L. , Read K. D. , and Fairlamb A. H. , Cross-Resistance to Nitro Drugs and Implications for Treatment of Human African Trypanosomiasis, Antimicrobial Agents and Chemotherapy. (2010) 54, no. 7, 2893–2900, 10.1128/aac.00332-10. 20439607 PMC2897277

[180] Torreele E. , Bourdin Trunz B. , Tweats D. et al., Fexinidazole–A New Oral Nitroimidazole Drug Candidate Entering Clinical Development for the Treatment of Sleeping Sickness, PLoS Neglected Tropical Diseases. (2010) 4, no. 12, 10.1371/journal.pntd.0000923. PMC3006138 21200426

[181] Docampo R. and Stoppani A. O. M. , Generation of Superoxide Anion and Hydrogen Peroxide Induced by Nifurtimox in Trypanosoma cruzi, Archives of Biochemistry and Biophysics. (1979) 197, no. 1, 317–321, 10.1016/0003-9861(79)90251-0. 232403

[182] Boiani M. , Piacenza L. , Hernández P. et al., Mode of Action of Nifurtimox and N-Oxide-Containing Heterocycles Against Trypanosoma cruzi: Is Oxidative Stress Involved?, Biochemical Pharmacology. (2010) 79, no. 12, 1736–1745, 10.1016/J.BCP.2010.02.009. 20178775

[183] Mejia A. M. , Hall B. S. , Taylor M. C. et al., Benznidazole-Resistance in Trypanosoma cruzi is a Readily Acquired Trait That Can Arise Independently in a Single Population, The Journal of Infectious Diseases. (2012) 206, no. 2, 220–228, 10.1093/infdis/jis331. 22551809 PMC3379838

[184] Milord F. , Pepin J. , Ethier L. , Loko L. , and Mpia B. , Efficacy and Toxicity of Eflornithine for Treatment of Trypanosoma brucei Gambiense Sleeping Sickness, The Lancet. (1992) 340, no. 8820, 652–655, 10.1016/0140-6736(92)92180-n. 1355219

[185] Vincent I. M. , Creek D. , Watson D. G. et al., A Molecular Mechanism for Eflornithine Resistance in African Trypanosomes, PLoS Pathogens. (2010) 6, no. 11, 10.1371/journal.ppat.1001204. PMC2991269 21124824

[186] Burkard G. S. , Jutzi P. , and Roditi I. , Genome-Wide RNAi Screens in Bloodstream Form Trypanosomes Identify Drug Transporters, Molecular and Biochemical Parasitology. (2011) 175, no. 1, 91–94, 10.1016/j.molbiopara.2010.09.002. 20851719

[187] Fischer J. , Nel-Son S. M. , Gleason K. G. et al., Alpha-Difluoromethylornithine, An Irreversible Inhibitor of Ornithine Decarboxylase, Inhibits Tumor Promoter-Induced Polyamine Accumulation and Carcinogenesis in Mouse Skin, Proceedings of the National Academy of Sciences of the United States of America. (1982) 79, no. 19, 10.1073/PNAS.79.19.6028. PMC347045 6821130

[188] Willert E. K. and Phillips M. A. , Regulated Expression of an Essential Allosteric Activator of Polyamine Biosynthesis in African Trypanosomes, PLoS Pathogens. (2008) 4, no. 10, 10.1371/journal.ppat.1000183. PMC2562514 18949025

[189] Giffin B. F. , Mccann P. P. , Bitonti A. J. , and Bacchi C. J. , Polyamine Depletion Following Exposure to DL‐α‐Difluoromethylornithine Both In Vivo and In Vitro Initiates Morphological Alterations and Mitochondrial Activation in a Monomorphic Strain of Trypanosoma brucei Brucei, Journal of Protozoology. (1986) 33, no. 2, 238–243, 10.1111/j.1550-7408.1986.tb05599.x. 3090240

[190] Zoltner M. , Leung K. F. , Alsford S. , Horn D. , and Field M. C. , Modulation of the Surface Proteome Through Multiple Ubiquitylation Pathways in African Trypanosomes, PLoS Pathogens. (2015) 11, no. 10, 10.1371/journal.ppat.1005236. PMC4619645 26492041

[191] Fairlamb A. H. and Bowman I. B. R. , Uptake of the Trypanocidal Drug Suramin by Bloodstream Forms of Trypanosoma brucei and its Effect on Respiration and Growth Rate In Vivo, Molecular and Biochemical Parasitology. (1980) 1, no. 6, 315–333, 10.1016/0166-6851(80)90050-x. 6108510

[192] Chello P. L. and Jaffe J. J. , Comparative Properties of Trypanosomal and Mammalian Thymidine Kinases, Comparative Biochemistry and Physiology Part B: Comparative Biochemistry. (1972) 43, no. 3, 543–562, 10.1016/0305-0491(72)90138-1. 4539354

[193] Jaffe J. J. , McCormack J. J. , and Meymarian E. , Comparative Properties of Schistosomal and Filarial Dihydrofolate Reductases, Biochemical Pharmacology. (1972) 21, no. 5, 719–731, 10.1016/0006-2952(72)90064-0. 4401788

[194] Leach T. M. and Roberts C. J. , Present Status of Chemotherapy and Chemoprophylaxis of Animal Trypanosomiasis in the Eastern Hemisphere, Pharmacology & Therapeutics. (1981) 13, no. 1, 91–147, 10.1016/0163-7258(81)90069-3. 7022488

[195] Sutherland I. A. , Mounsey A. , and Holmes P. H. , Transport of Isometamidium (Samorin) by Drug-Resistant and Drug-Sensitive Trypanosoma congolense, Parasitology. (1992) 104, no. 3, 461–467, 10.1017/s0031182000063721. 1641246

[196] De Koning H. P. , Bridges D. J. , and Burchmore R. J. S. , Purine and Pyrimidine Transport in Pathogenic Protozoa: From Biology to Therapy, FEMS Microbiology Reviews. (2005) 29, no. 5, 987–1020, 10.1016/J.FEMSRE.2005.03.004. 16040150

[197] Dougherty G. and Waring M. J. , The Interaction Between Prothidium Dibromide and DNA at the Molecular Level, Biophysical Chemistry. (1982) 15, no. 1, 27–40, 10.1016/0301-4622(82)87014-2. 7074206

[198] Sutherland I. A. , Peregrine A. S. , Lonsdale-Eccles J. D. , and Holmes P. H. , Reduced Accumulation of Isometamidium by Drug-Resistant Trypanosoma Congolense, Parasitology. (1991) 103, no. 2, 245–251, 10.1017/s0031182000059527. 1745550

[199] Tihon E. , Imamura H. , Van den Broeck F. , Vermeiren L. , Dujardin J.-C. , and Van Den Abbeele J. , Genomic Analysis of Isometamidium Chloride Resistance in Trypanosoma Congolense, International Journal of Parasitology: Drugs and Drug Resistance. (2017) 7, no. 3, 350–361, 10.1016/j.ijpddr.2017.10.002. PMC5645165 29032180

[200] Wilkes J. M. , Mulugeta W. , Wells C. , and Peregrine A. S. , Modulation of Mitochondrial Electrical Potential: A Candidate Mechanism for Drug Resistance in African Trypanosomes, Biochemical Journal. (1997) 326, no. 3, 755–761, 10.1042/bj3260755. 9307025 PMC1218730

[201] Moloo S. K. and Kutuza S. B. , Expression of Resistance to Isometamidium and Diminazene in Trypanosoma Congolense in Boran Cattle Infected by Glossina morsitans centralis, Acta Tropica. (1990) 47, no. 2, 79–89, 10.1016/0001-706x(90)90070-g. 1969704

[202] Peregrine A. S. , Gray M. A. , and Moloo S. K. , Cross-Resistance Associated With Development of Resistance to Isometamidium in a Clone of Trypanosoma congolense, Antimicrobial Agents and Chemotherapy. (1997) 41, no. 7, 1604–1606, 10.1128/aac.41.7.1604. 9210695 PMC163969

[203] Lanteri C. A. , Stewart M. L. , Brock J. M. et al., Roles for the Trypanosoma brucei P2 Transporter in DB75 Uptake and Resistance, Molecular Pharmacology. (2006) 70, no. 5, 1585–1592, 10.1124/mol.106.024653. 16912218

[204] Jones D. C. , Foth B. J. , Urbaniak M. D. et al., Genomic and Proteomic Studies on the Mode of Action of Oxaboroles Against the African Trypanosome, PLoS Neglected Tropical Diseases. (2015) 9, no. 12, 1–18, 10.1371/journal.pntd.0004299. PMC4689576 26684831

[205] Nagle A. , Biggart A. , Be C. et al., Discovery and Characterization of Clinical Candidate LXE408 as a Kinetoplastid-Selective Proteasome Inhibitor for the Treatment of Leishmaniases, Journal of Medicinal Chemistry. (2020) 63, no. 19, 10773–10781, 10.1021/acs.jmedchem.0c00499. 32667203 PMC7549094

[206] Thuita J. K. , Karanja S. M. , Wenzler T. et al., Efficacy of the Diamidine DB75 and its Prodrug DB289, Against Murine Models of Human African Trypanosomiasis, Acta Tropica. (2008) 108, no. 1, 6–10, 10.1016/j.actatropica.2008.07.006. 18722336

[207] Pohlig G. , Bernhard S. C. , Blum J. et al., Efficacy and Safety of Pafuramidine versus Pentamidine Maleate for Treatment of First Stage Sleeping Sickness in a Randomized, Comparator-Controlled, International Phase 3 Clinical Trial, PLoS Neglected Tropical Diseases. (2016) 10, no. 2, 1–17, 10.1371/journal.pntd.0004363. PMC4755561 26882015

[208] Mathis A. M. , Holman J. L. , Sturk L. M. et al., Accumulation and Intracellular Distribution of Antitrypanosomal Diamidine Compounds DB75 and DB820 in African Trypanosomes, Antimicrobial Agents and Chemotherapy. (2006) 50, no. 6, 2185–2191, 10.1128/AAC.00192-06. 16723581 PMC1479144

[209] Paine M. F. , Wang M. Z. , Generaux C. N. et al., Diamidines for Human African Trypanosomiasis, Current Opinion in Investigational Drugs. (2010) 11, no. 8, 876–883. 20721830

[210] Lanteri C. A. , Tidwell R. R. , and Meshnick S. R. , The Mitochondrion is a Site of Trypanocidal Action of the Aromatic Diamidine DB75 in Bloodstream Forms of Trypanosoma brucei, Antimicrobial Agents and Chemotherapy. (2008) 52, no. 3, 875–882, 10.1128/AAC.00642-07. 18086841 PMC2258549

[211] Zuma A. A. , Cavalcanti D. P. , Zogovich M. et al., Unveiling the Effects of Berenil, A DNA-Binding Drug, on Trypanosoma cruzi: Implications for kDNA Ultrastructure and Replication, Parasitology Research. (2015) 114, no. 2, 419–430, 10.1007/S00436-014-4199-8. 25349143

[212] Ding D. , Zhao Y. , Meng Q. et al., Discovery of Novel Benzoxaborole-Based Potent Antitrypanosomal Agents, ACS Medicinal Chemistry Letters. (2010) 1, no. 4, 165–169, 10.1021/ml100013s. 24900190 PMC4007846

[213] Jacobs R. T. , Platter J. J. , Nare B. et al., Benzoxaboroles: A New Class of Potential Drugs for Human African Trypanosomiasis, Future Medicinal Chemistry. (2011) 3, no. 10, 1259–1278, 10.4155/fmc.11.80. 21859301

[214] Kumeso V. K. B. , Kalonji W. M. , Rembry S. et al., Efficacy and Safety of Acoziborole in Patients With Human African Trypanosomiasis Caused by Trypanosoma brucei gambiense: A Multicentre, Open-Label, Single-Arm, Phase 2/3 Trial, The Lancet Infectious Diseases. (2023) 23, no. 4, 463–470, 10.1016/S1473-3099(22)00660-0. 36460027 PMC10033454

[215] Waithaka A. and Clayton C. , Clinically Relevant Benzoxaboroles Inhibit mRNA Processing in Trypanosoma brucei, BMC Research Notes. (2022) 15, no. 1, 1–6, 10.1186/s13104-022-06258-y. 36528767 PMC9758897

[216] Wall R. J. , Rico E. , Lukac I. et al., Clinical and Veterinary Trypanocidal Benzoxaboroles Target CPSF3, Proceedings of the National Academy of Sciences of the United States of America. (2018) 115, no. 38, 9616–9621, 10.1073/pnas.1807915115. 30185555 PMC6156652

[217] Drugs for Neglected Diseases Initiative DND , AN2 Therapeutics and DNDi Collaborate on Clinical Development of Promising New Oral Compound to Treat Chronic Chagas Disease, 2025, https://dndi.org/press-releases/2025/an2-therapeutics-and-dndi-collaborate-on-clinical-development-of-promising-new-oral-compound-to-treat-chronic-chagas-disease/.

[218] Drugs for Neglected Diseases Initiative DND , LXE408 Novartis for Cutaneous Leishmaniasis| DNDi, 2025, https://dndi.org/research-development/portfolio/lxe408-novartis-for-cutaneous-leishmaniasis/.

[219] Santucci M. , Luciani R. , Gianquinto E. et al., Repurposing the Trypanosomatidic GSK Kinetobox for the Inhibition of Parasitic Pteridine and Dihydrofolate Reductases, Pharmaceuticals. (2021) 14, no. 12, 10.3390/ph14121246. PMC8704748 34959646

[220] Linciano P. , Dawson A. , Pöhner I. et al., Exploiting the 2-Amino-1, 3, 4-Thiadiazole Scaffold to Inhibit Trypanosoma brucei Pteridine Reductase in Support of Early-Stage Drug Discovery, ACS Omega. (2017) 2, no. 9, 5666–5683, 10.1021/acsomega.7b00473. 28983525 PMC5623949

[221] Prieto Barja P. , Pescher P. , Bussotti G. et al., Haplotype Selection as an Adaptive Mechanism in the Protozoan Pathogen Leishmania donovani, Nature Ecology & Evolution. (2017) 1, no. 12, 1961–1969, 10.1038/s41559-017-0361-x. 29109466

[222] Bussotti G. , Gouzelou E. , Côrtes Boité M. et al., Leishmania Genome Dynamics During Environmental Adaptation Reveal Strain-Specific Differences in Gene Copy Number Variation, Karyotype Instability, and Telomeric Amplification, mBio. (2018) 9, no. 6, 10–1128, 10.1128/mbio.01399-18. PMC6222132 30401775

[223] Mandell M. A. and Beverley S. M. , Continual Renewal and Replication of Persistent Leishmania Major Parasites in Concomitantly Immune Hosts, Proceedings of the National Academy of Sciences. (2017) 114, no. 5, E801–E810, 10.1073/pnas.1619265114. PMC5293024 28096392

[224] Dumetz F. , Cuypers B. , Imamura H. et al., Molecular Preadaptation to Antimony Resistance in Leishmania donovani on the Indian Subcontinent, mSphere. (2018) 3, no. 2, 10–1128, 10.1128/msphere.00548-17. PMC5907651 29669889

[225] Dehoux J. P. , Chemoprevention of Bovine Trypanosomiasis in N’Dama Cattle Imported From the Senegambia and Zaire to Gabon, Revue D’élevage et de Médecine Vétérinaire Des Pays Tropicaux. (1990) 43, no. 3, 337–341. 2103056

[226] Aregawi W. G. , Gutema F. , Tesfaye J. et al., Efficacy of Diminazene Diaceturate and Isometamidium Chloride Hydrochloride for the Treatment of Trypanosoma Evansi in Mice Model, Journal of Parasitic Diseases. (2021) 45, no. 1, 131–136, 10.1007/s12639-020-01289-3. 33746398 PMC7921223

[227] Magona J. W. , Mayende J. S. P. , Okiria R. , and Okuna N. M. , Protective Efficacy of Isometamidium Chloride and Diminazene Aceturate against Natural Trypanosoma brucei, Trypanosoma congolense and Trypanosoma vivax Infections in Cattle Under a Suppressed Tsetse Population in Uganda, Onderstepoort Journal of Veterinary Research. (2004) 71, no. 3, 231–237. 15580773 10.4102/ojvr.v71i3.265

[228] Ihedioha J. I. , Ochiogu I. S. , and Ihedioha T. E. , Co-Administration of Na-EDTA and Diminazene Aceturate (DA) to Mice Infected With DA-Resistant Trypanosoma brucei, Journal of Comparative Pathology. (2007) 136, no. 2–3, 206–211, 10.1016/j.jcpa.2007.01.003. 17367805

[229] Dina O. A. , Saba A. B. , Adedapo A. A. , Akinyemi O. A. , and Ombowale T. O. , Comparative Efficacy Study of Homidium Bromde, Diminazene Aceturate and Their Combination in New Zealand White Rabbits Experimentally Infected With Trypanosoma Congolense, Tropical Veterinarian. (2002) 20, no. 3, 153–158, 10.4314/tv.v20i3.4495.

[230] Zhang Z. Q. , Giroud C. , and Baltz T. , Trypanosoma Evansi: In Vivo and In Vitro Determination of Trypanocide Resistance Profiles, Experimental Parasitology. (1993) 77, no. 4, 387–394, 10.1006/expr.1993.1098. 8253152

[231] Anene B. M. , Ross C. A. , Anika S. M. , and Chukwu C. C. , Trypanocidal Resistance in Trypanosoma Evansi In Vitro: Effects of Verapamil, Cyproheptidine, Desipramine and Chlorpromazine Alone and in Combination With Trypanocides, Veterinary Parasitology. (1996) 62, no. 1–2, 43–50, 10.1016/0304-4017(95)00856-x. 8638392

[232] Priotto G. , Kasparian S. , Mutombo W. et al., Nifurtimox-Eflornithine Combination Therapy for Second-Stage African Trypanosoma brucei Gambiense Trypanosomiasis: A Multicentre, Randomised, Phase III, Non-Inferiority Trial, The Lancet. (2009) 374, no. 9683, 56–64, 10.1016/s0140-6736(09)61117-x. 19559476

[233] Goswami R. P. , Rahman M. , Das S. , Tripathi S. K. , and Goswami R. P. , Combination Therapy Against Indian Visceral Leishmaniasis With Liposomal Amphotericin B (FungisomeTM) and Short-Course Miltefosine in Comparison to Miltefosine Monotherapy, The American Journal of Tropical Medicine and Hygiene. (2020) 103, no. 1, 308–314, 10.4269/ajtmh.19-0931. 32394874 PMC7356435

[234] Tabrez S. , Rahman F. , Ali R. et al., Repurposing of FDA‐Approved Drugs as Inhibitors of Sterol C‐24 Methyltransferase of Leishmania donovani to Fight Against Leishmaniasis, Drug Development Research. (2021) 82, no. 8, 1154–1161, 10.1002/ddr.21820. 33929761

[235] Babokhov P. , Sanyaolu A. O. , Oyibo W. A. , Fagbenro-Beyioku A. F. , and Iriemenam N. C. , A Current Analysis of Chemotherapy Strategies for the Treatment of Human African Trypanosomiasis, Pathogens and Global Health. (2013) 107, no. 5, 242–252, 10.1179/2047773213y.0000000105. 23916333 PMC4001453

[236] Tekle T. , Terefe G. , Cherenet T. et al., Aberrant Use and Poor Quality of Trypanocides: A Risk for Drug Resistance in South Western Ethiopia, BMC Veterinary Research. (2018) 14, 1–8, 10.1186/s12917-017-1327-6. 29304792 PMC5755418

[237] Shaw A. P. M. , Tirados I. , Mangwiro C. T. N. et al., Costs of Using “Tiny Targets” to Control Glossina fuscipes fuscipes, A Vector of Gambiense Sleeping Sickness in Arua District of Uganda, PLoS Neglected Tropical Diseases. (2015) 9, no. 3, 10.1371/journal.pntd.0003624. PMC4374750 25811956

[238] Kulohoma B. W. , Wamwenje S. A. O. , Wangwe I. I. , Masila N. , Mirieri C. K. , and Wambua L. , Prevalence of Trypanosomes Associated With Drug Resistance in Shimba Hills, Kwale County, Kenya, BMC Research Notes. (2020) 13, 1–6, 10.1186/s13104-020-05077-3. 32349785 PMC7191804

[239] Magez S. , Li Z. , Nguyen H. T. T. et al., The History of Anti-Trypanosome Vaccine Development Shows That Highly Immunogenic and Exposed Pathogen-Derived Antigens Are Not Necessarily Good Target Candidates: Enolase and ISG75 as Examples, Pathogens. (2021) 10, no. 8, 10.3390/pathogens10081050. PMC8400590 34451514

[240] Autheman D. , Crosnier C. , Clare S. et al., An Invariant Trypanosoma vivax Vaccine Antigen Induces Protective Immunity, Nature. (2021) 595, no. 7865, 96–100, 10.1038/s41586-021-03597-x. 34040257

[241] Ortiz D. , Sanchez M. A. , Quecke P. , and Landfear S. M. , Two Novel Nucleobase/Pentamidine Transporters From Trypanosoma brucei, Molecular and Biochemical Parasitology. (2009) 163, no. 2, 67–76, 10.1016/j.molbiopara.2008.09.011. 18992774 PMC2630410

[242] Salari S. , Bamorovat M. , Sharifi I. , and Almani P. G. N. , Global Distribution of Treatment Resistance Gene Markers for Leishmaniasis, Journal of Clinical Laboratory Analysis. (2022) 36, no. 8, 10.1002/jcla.24599. PMC9396204 35808933

[243] Simo G. , Magang E. M. K. , Mewamba E. M. et al., Molecular Identification of Diminazene Aceturate Resistant Trypanosomes in Tsetse Flies From Yoko in the Centre Region of Cameroon and its Epidemiological Implications, Parasite Epidemiology and Control. (2020) 9, 10.1016/j.parepi.2020.e00135. PMC6957779 31956704

[244] Veepanattu P. , Singh S. , Mendelson M. et al., Building Resilient and Responsive Research Collaborations to Tackle Antimicrobial Resistance—Lessons Learnt From India, South Africa, and UK, International Journal of Infectious Diseases. (2020) 100, 278–282, 10.1016/j.ijid.2020.08.057. 32860949 PMC7449941

[245] Kuemmerle A. , Schmid C. , Bernhard S. et al., Effectiveness of Nifurtimox Eflornithine Combination Therapy (NECT) in T. b. Gambiense Second Stage Sleeping Sickness Patients in the Democratic Republic of Congo: Report From a Field Study, PLoS Neglected Tropical Diseases. (2021) 15, no. 11, 10.1371/journal.pntd.0009903. PMC8601604 34748572

[246] Bisser S. , N’Siesi F.-X. , Lejon V. et al., Equivalence Trial of Melarsoprol and Nifurtimox Monotherapy and Combination Therapy for the Treatment of Second-Stage Trypanosoma brucei gambiense Sleeping Sickness, The Journal of Infectious Diseases. (2007) 195, no. 3, 322–329, 10.1086/510534. 17205469

[247] Mulugeta W. , Wilkes J. , Mulatu W. , Majiwa P. A. O. , Masake R. , and Peregrine A. S. , Long-term Occurrence of Trypanosoma congolense Resistant to Diminazene, Isometamidium and Homidium in Cattle at Ghibe, Ethiopia, Acta Tropica. (1997) 64, no. 3–4, 205–217, 10.1016/s0001-706x(96)00645-6. 9107367

[248] Mungube E. O. , Vitouley H. S. , Allegye-Cudjoe E. et al., Detection of Multiple Drug-Resistant Trypanosoma congolense Populations in Village Cattle of South-East Mali, Parasites & Vectors. (2012) 5, 1–9, 10.1186/1756-3305-5-155. 22852796 PMC3432589

[249] Barrett M. P. , Vincent I. M. , Burchmore R. J. S. , Kazibwe A. J. N. , and Matovu E. , Drug Resistance in Human African Trypanosomiasis, Future Microbiology. (2011) 6, no. 9, 1037–1047, 10.2217/fmb.11.88. 21958143

[250] Sundar S. , Sinha P. K. , Rai M. et al., Comparison of Short-Course Multidrug Treatment With Standard Therapy for Visceral Leishmaniasis in India: An Open-Label, Non-Inferiority, Randomised Controlled Trial, The Lancet. (2011) 377, no. 9764, 477–486, 10.1016/s0140-6736(10)62050-8. 21255828

[251] Rahman R. , Goyal V. , Haque R. et al., Safety and Efficacy of Short Course Combination Regimens With AmBisome, Miltefosine and Paromomycin for the Treatment of Visceral Leishmaniasis (VL) in Bangladesh, PLoS Neglected Tropical Diseases. (2017) 11, no. 5, 10.1371/journal.pntd.0005635. PMC5466346 28558062

[252] Clausen P. , Bauer B. , Zessin K. et al., Preventing and Containing Trypanocide Resistance in the Cotton Zone of West Africa, Transboundary and Emerging Diseases. (2010) 57, no. 1‐2, 28–32, 10.1111/j.1865-1682.2010.01129.x. 20537098

[253] Van den Bossche P. , Doran M. , and Connor R. J. , An Analysis of Trypanocidal Drug Use in the Eastern Province of Zambia, Acta Tropica. (2000) 75, no. 2, 247–258, 10.1016/s0001-706x(00)00059-0. 10708665

[254] Shiferaw S. , Muktar Y. , and Belina D. , A Review on Trypanocidal Drug Resistance in Ethiopia, Journal of Parasitology and Vector Biology. (2015) 7, no. 4, 58–66.

[255] Kasozi K. I. , MacLeod E. T. , Ntulume I. , and Welburn S. C. , An Update on African Trypanocide Pharmaceutics and Resistance, Frontiers in Veterinary Science. (2022) 9, 10.3389/fvets.2022.828111. PMC8959112 35356785

[256] Tweats D. , Bourdin Trunz B. , and Torreele E. , Genotoxicity Profile of Fexinidazole—A Drug Candidate in Clinical Development for Human African Trypanomiasis (Sleeping Sickness), Mutagenesis. (2012) 27, no. 5, 523–532, 10.1093/mutage/ges015. 22539226

[257] Maser P. , Wittin S. , Rottmann M. , Wenzler T. , Kaiser M. , and Brun R. , Antiparasitic Agents: New Drugs on the Horizon, Current Opinion in Pharmacology. (2012) 12, no. 5, 562–566. 22652215 10.1016/j.coph.2012.05.001

[258] Steinmann P. , Stone C. M. , Sutherland C. S. , Tanner M. , and Tediosi F. , Contemporary and Emerging Strategies for Eliminating Human African Trypanosomiasis Due to Trypanosoma brucei Gambiense, Tropical Medicine and International Health. (2015) 20, no. 6, 707–718, 10.1111/tmi.12483. 25694261

[259] Boitz J. M. , Ullman B. , Jardim A. , and Carter N. S. , Purine Salvage in Leishmania: Complex or Simple by Design?, Trends in Parasitology. (2012) 28, no. 8, 345–352, 10.1016/j.pt.2012.05.005. 22726696 PMC3429121

[260] Venturelli A. , Tagliazucchi L. , Lima C. et al., Current Treatments to Control African Trypanosomiasis and One Health Perspective, Microorganisms. (2022) 10, no. 7, 10.3390/microorganisms10071298. PMC9321528 35889018

[261] Aoki J. I. , Coelho A. C. , Muxel S. M. et al., Characterization of a Novel Endoplasmic Reticulum Protein Involved in Tubercidin Resistance in Leishmania Major, PLoS Neglected Tropical Diseases. (2016) 10, no. 9, 10.1371/journal.pntd.0004972. PMC5015992 27606425

[262] Vodnala S. K. , Lundbäck T. , Yeheskieli E. et al., Structure–Activity Relationships of Synthetic Cordycepin Analogues as Experimental Therapeutics for African Trypanosomiasis, Journal of Medicinal Chemistry. (2013) 56, no. 24, 9861–9873, 10.1021/jm401530a. 24283924

[263] Pena I. , Pilar Manzano M. , Cantizani J. et al., New Compound Sets Identified From High Throughput Phenotypic Screening Against Three Kinetoplastid Parasites: An Open Resource, Scientific Reports. (2015) 5, no. 1, 10.1038/srep08771. PMC4350103 25740547

[264] Kumari D. , Perveen S. , Sharma R. , and Singh K. , Advancement in Leishmaniasis Diagnosis and Therapeutics: An Update, European Journal of Pharmacology. (2021) 910, 10.1016/j.ejphar.2021.174436. 34428435

[265] Lima M. L. , Abengózar M. A. , Nácher-Vázquez M. et al., Molecular Basis of the Leishmanicidal Activity of the Antidepressant Sertraline as a Drug Repurposing Candidate, Antimicrobial Agents and Chemotherapy. (2018) 62, no. 12, 10–1128, 10.1128/aac.01928-18. PMC6256786 30297370

[266] Khadir F. , Taheri T. , Habibzadeh S. et al., Antileishmanial Effect of Rapamycin as an Alternative Approach to Control Leishmania tropica Infection, Veterinary Parasitology. (2019) 276, 10.1016/j.vetpar.2019.108976. 31739256

[267] Keyhani A. , Jafarzadeh A. , Sharifi I. , and Salarkia E. , Leptin Enhances the Efficacy of Glucantime to Modulate Macrophage Polarization Toward the M1 Phenotype in *Leishmania tropica*-Infected Macrophages, Parasites & Vectors. (2025) 18, no. 1, 10.1186/s13071-025-07004-6. PMC12379411 40855340

[268] Kyriazis I. D. , Koutsoni O. S. , Aligiannis N. , Karampetsou K. , Skaltsounis A.-L. , and Dotsika E. , The Leishmanicidal Activity of Oleuropein is Selectively Regulated through Inflammation-And Oxidative Stress-Related Genes, Parasites & Vectors. (2016) 9, 1–16, 10.1186/s13071-016-1701-4. 27501956 PMC4977900

[269] Parihar S. , Hartley M.-A. , Hurdayal R. , Guler R. , and Brombacher F. , Topical Simvastatin as Host-Directed Therapy Against Severity of Cutaneous Leishmaniasis in Mice, Scientific Reports. (2016) 16, no. 6, 10.1038/srep33458. PMC5025842 27632901

[270] Alsford S. A. M. , Kelly J. M. , Baker N. , and Horn D. , Genetic Dissection of Drug Resistance in Trypanosomes, Parasitology. (2013) 140, no. 12, 1478–1491, 10.1017/s003118201300022x. 23552488 PMC3759293

[271] Simarro P. P. , Diarra A. , Ruiz Postigo J. A. , Franco J. R. , and Jannin J. G. , The Human African Trypanosomiasis Control and Surveillance Programme of the World Health Organization 2000–2009: the Way Forward, PLoS Neglected Tropical Diseases. (2011) 5, no. 2, 10.1371/journal.pntd.0001007. PMC3042999 21364972

[272] Matovu E. , Seebeck T. , Enyaru J. C. , and Kaminsky R. , Drug Resistance in Trypanosoma brucei spp., the Causative Agents of Sleeping Sickness in Man and Nagana in Cattle, Microbes and Infection. (2001) 3, no. 9, 763–770, 10.1016/s1286-4579(01)01432-0. 11489425

[273] Gibson W. and Whittington H. , Genetic Exchange in Trypanosoma brucei: Selection of Hybrid Trypanosomes by Introduction of Genes Conferring Drug Resistance, Molecular and Biochemical Parasitology. (1993) 60, no. 1, 19–26, 10.1016/0166-6851(93)90024-r. 8366892

[274] Aksoy S. , Buscher P. , Lehane M. , Solano P. , and Van Den Abbeele J. , Human African Trypanosomiasis Control: Achievements and Challenges, PLoS Neglected Tropical Diseases. (2017) 11, no. 4, 10.1371/journal.pntd.0005454. PMC5398477 28426685

[275] Naderer T. , Heng J. , Saunders E. C. et al., Intracellular Survival of Leishmania Major Depends on Uptake and Degradation of Extracellular Matrix Glycosaminoglycans by Macrophages, PLoS Pathogens. (2015) 11, no. 9, 10.1371/journal.ppat.1005136. PMC4559419 26334531

[276] Gazos-Lopes F. , Martin J. L. , Dumoulin P. C. , and Burleigh B. A. , Host Triacylglycerols Shape the Lipidome of Intracellular Trypanosomes and Modulate Their Growth, PLoS Pathogens. (2017) 13, no. 12, 10.1371/journal.ppat.1006800. PMC5760102 29281741

[277] Rojas F. and Matthews K. R. , Quorum Sensing in African Trypanosomes, Current Opinion in Microbiology. (2019) 52, 124–129, 10.1016/j.mib.2019.07.001. 31442903

[278] Rogers M. B. , Hilley J. D. , Dickens N. J. et al., Chromosome and Gene Copy Number Variation Allow Major Structural Change Between Species and Strains of Leishmania, Genome Research. (2011) 21, no. 12, 2129–2142, 10.1101/gr.122945.111. 22038252 PMC3227102

[279] Ferreira T. R. , Inbar E. , Shaik J. , Jeffrey B. M. , Ghosh K. , and Dobson D. E. , Self-hybridization in Leishmania Major, mBio. (2022) 13, no. 6, 10.1128/mbio.02858-22. PMC9764971 36394334

[280] González-Salazar C. , Meneses-Mosquera A. K. , Aguirre-Peña A. et al., Toward New Epidemiological Landscapes of Trypanosoma cruzi (Kinetoplastida, Trypanosomatidae) Transmission Under Future Human-Modified Land Cover and Climatic Change in Mexico, Tropical Medicine and Infectious Disease. (2022) 7, no. 9, 10.3390/tropicalmed7090221. PMC9503189 36136632

[281] Wamwenje S. A. O. , Wangwe I. I. , Masila N. , Mirieri C. K. , Wambua L. , and Kulohoma B. W. , Community-Led Data Collection Using Open Data Kit for Surveillance of Animal African Trypanosomiasis in Shimba Hills, Kenya, BMC Research Notes. (2019) 12, 1–6, 10.1186/s13104-019-4198-z. 30885271 PMC6423862

[282] Dinc R. , Leishmania Vaccines: The Current Situation With its Promising Aspect for the Future, Korean Journal of Parasitology. (2022) 60, no. 6, 379–391, 10.3347/kjp.2022.60.6.379. 36588414 PMC9806502

[283] de Oliveira D. S. , Zaldívar M. F. , Gonçalves A. A. M. et al., New Approaches to the Prevention of Visceral Leishmaniasis: A Review of Recent Patents of Potential Candidates for a Chimeric Protein Vaccine, Vaccines. (2024) 12, no. 3, 10.3390/vaccines12030271. PMC10975552 38543905

[284] Rajaraman S. , Antani S. K. , Poostchi M. et al., Pre-Trained Convolutional Neural Networks as Feature Extractors Toward Improved Malaria Parasite Detection in Thin Blood Smear Images, PeerJ. (2018) 6, 10.7717/peerj.4568. PMC5907772 29682411

[285] Morais M. C. C. , Silva D. , Milagre M. M. et al., Automatic Detection of the Parasite Trypanosoma cruzi in Blood Smears Using a Machine Learning Approach Applied to Mobile Phone Images, PeerJ. (2022) 10, 10.7717/peerj.13470. PMC9150695 35651746

[286] Abdelmula A. M. , Mirzaei O. , Güler E. , and Süer K. , Assessment of Deep Learning Models for Cutaneous Leishmania Parasite Diagnosis Using Microscopic Images, Diagnostics. (2024) 14, no. Issue 1, 10.3390/diagnostics14010012. PMC10802189 38201321

[287] Ronneberger O. , Fischer P. , and Brox T. , U-Net: Convolutional Networks for Biomedical Image Segmentation, *Medical Image Computing and Computer-Assisted Intervention–MICCAI 2015: 18th International Conference*, October 5-9, 2015, Munich, Germany.

[288] Górriz M. , Aparicio A. , Raventós B. , Vilaplana V. , Sayrol E. , and López-Codina D. , Perales F. J. and Kittler J. , Leishmaniasis Parasite Segmentation and Classification Using Deep Learning BT-Articulated Motion and Deformable Objects, 2018, Springer International Publishing, 53–62.

[289] Sanchez-Patiño N. , Toriz-Vazquez A. , Hevia-Montiel N. , and Perez-Gonzalez J. , Convolutional Neural Networks for Chagas’ Parasite Detection in Histopathological Images, *2021 43rd Annual International Conference of the IEEE Engineering in Medicine & Biology Society (EMBC)*, 2021, 2732–2735, 10.1109/EMBC46164.2021.9629563. 34891815

[290] Hosny K. M. , Kassem M. A. , and Foaud M. M. , Classification of Skin Lesions Using Transfer Learning and Augmentation With Alex-Net, PLoS One. (2019) 14, no. 5, 10.1371/journal.pone.0217293. PMC6529006 31112591

[291] Leal J. F. , Barroso D. H. , Trindade N. S. , Miranda V. L. , and Gurgel-Gonçalves R. , Automated Identification of Cutaneous Leishmaniasis Lesions Using Deep-Learning-Based Artificial Intelligence, Biomedicines. (2024) 12, no. 1, 10.3390/biomedicines12010012. PMC10813291 38275373

[292] Pereira A. S. , Mazza L. O. , Pinto P. C. C. et al., Deep Convolutional Neural Network Applied to Trypanosoma cruzi Detection in Blood Samples, International Journal of Bio-Inspired Computation. (2022) 19, no. 1, 1–17, 10.1504/ijbic.2022.120749.

[293] Howard A. , Sandler M. , Chu G. et al., Searching for Mobilenetv3, Proceedings of the IEEE/CVF International Conference on Computer Vision. (2019) 1314–1324.

[294] Zare M. , Akbarialiabad H. , Parsaei H. et al., A Machine Learning-Based System for Detecting Leishmaniasis in Microscopic Images, BMC Infectious Diseases. (2022) 22, no. 1, 10.1186/s12879-022-07029-7. PMC8754077 35022031

[295] Oliveira A. D. , Prats C. , Espasa M. et al., The Malaria System MicroApp: A New, Mobile Device-Based Tool for Malaria Diagnosis, JMIR Research Protocols. (2017) 6, no. 4, 10.2196/resprot.6758. PMC5424126 28442456

[296] Uc-Cetina V. , Brito-Loeza C. , and Ruiz-Piña H. , Chagas Parasite Detection in Blood Images Using AdaBoost, Computational and Mathematical Methods in Medicine. (2015) 2015, no. 1, 139681–13, 10.1155/2015/139681. 25861375 PMC4377374

[297] Zhang C. , Jiang H. , Jiang H. et al., Deep Learning for Microscopic Examination of Protozoan Parasites, Computational and Structural Biotechnology Journal. (2022) 20, 1036–1043, 10.1016/j.csbj.2022.02.005. 35284048 PMC8886013

[298] Jomtarak R. , Kittichai V. , Kaewthamasorn M. et al., Mobile Bot Application for Identification of Trypanosoma Evansi Infection through Thin-Blood Film Examination Based on Deep Learning Approach, *2023 IEEE International Conference on Cybernetics and Innovations (ICCI)*, 2023, 1–7, 10.1109/ICCI57424.2023.10112327.

[299] Kahraman İ. , Karaş İ. R. , and Turan M. K. , Real-Time Protozoa Detection From Microscopic Imaging Using YOLOv4 Algorithm, Applied Sciences. (2024) 14, no. 2, 10.3390/app14020607.

[300] Kittichai V. , Kaewthamasorn M. , Thanee S. et al., Superior Auto-Identification of Trypanosome Parasites by Using a Hybrid Deep-Learning Model, Journal of Visualized Experiments. (2023) 200, 10.3791/65557. 37955392

[301] Fairlamb A. H. and Wyllie S. , The Critical Role of Mode of Action Studies in Kinetoplastid Drug Discovery, Frontiers in Drug Discovery. (2023) 3, 10.3389/fddsv.2023.1185679. PMC7614965 37600222

[302] Chen X. and Ishwaran H. , Random Forests for Genomic Data Analysis, Genomics. (2012) 99, no. 6, 323–329, 10.1016/j.ygeno.2012.04.003. 22546560 PMC3387489

[303] Breiman L. , Random Forests, Machine Learning. (2001) 45, no. 1, 5–32, 10.1023/A:1010933404324.

[304] Ali T. , Ahmed S. , and Aslam M. , Artificial Intelligence for Antimicrobial Resistance Prediction: Challenges and Opportunities Towards Practical Implementation, Antibiotics. (2023) 12, no. 3, 10.3390/antibiotics12030523. PMC10044311 36978390

[305] O’Boyle N. M. , Banck M. , James C. A. , Morley C. , Vandermeersch T. , and Hutchison G. R. , Open Babel: An Open Chemical Toolbox, Journal of Cheminformatics. (2011) 3, 1–14, 10.1186/1758-2946-3-33. 21982300 PMC3198950

[306] Eberhardt J. , Santos-Martins D. , Tillack A. F. , and Forli S. , AutoDock Vina 1.2. 0: New Docking Methods, Expanded Force Field, and Python Bindings, Journal of Chemical Information and Modeling. (2021) 61, no. 8, 3891–3898, 10.1021/acs.jcim.1c00203. 34278794 PMC10683950

[307] Reigada C. , Valera-Vera E. A. , Sayé M. et al., Trypanocidal Effect of Isotretinoin Through the Inhibition of Polyamine and Amino Acid Transporters in Trypanosoma cruzi, PLoS Neglected Tropical Diseases. (2017) 11, no. 3, 10.1371/journal.pntd.0005472. PMC5371382 28306713

[308] Reigada C. , Sayé M. , Phanstiel IV O. , Valera-Vera E. , Miranda M. R. , and Pereira C. A. , Identification of Trypanosoma cruzi Polyamine Transport Inhibitors by Computational Drug Repurposing, Frontiers of Medicine. (2019) 6, 10.3389/fmed.2019.00256. PMC6857147 31781568

